# Single-cell RNA sequencing of cervical exfoliated cells reveals potential biomarkers and cellular pathogenesis in cervical carcinogenesis

**DOI:** 10.1038/s41419-024-06522-y

**Published:** 2024-02-12

**Authors:** Bo Sheng, Shuya Pan, Miaomiao Ye, Hejing Liu, Jiamin Zhang, Bo Zhao, Huihui Ji, Xueqiong Zhu

**Affiliations:** Zhejiang Provincial Clinical Research Center for Obstetrics and Gynecology, Department of Obstetrics and Gynecology, The Second Affiliated Hospital of Wenzhou Medical University, Wenzhou, 325027 China

**Keywords:** Cervical cancer, Cancer microenvironment

## Abstract

Cervical cancer (CC) is a common gynecological malignancy. Despite the current screening methods have been proved effectively and significantly decreased CC morbidity and mortality, deficiencies still exist. Single-cell RNA sequencing (scRNA-seq) approach can identify the complex and rare cell populations at single-cell resolution. By scRNA-seq, the heterogeneity of tumor microenvironment across cervical carcinogenesis has been mapped and described. Whether these alterations could be detected and applied to CC screening is unclear. Herein, we performed scRNA-seq of 56,173 cervical exfoliated cells from 15 samples, including normal cervix, low-grade squamous intraepithelial lesion (LSIL), high-grade squamous intraepithelial lesion (HSIL), and malignancy. The present study delineated the alteration of immune and epithelial cells derived during the cervical lesion progression. A subset of lipid-associated macrophage was identified as a tumor-promoting element and could serve as a biomarker for predicting the progression of LSIL into HSIL, which was then verified by immunofluorescence. Furthermore, cell–cell communication analysis indicated the *SPP1-CD44* axis might exhibit a protumor interaction between epithelial cell and macrophage. In this study, we investigated the cervical multicellular ecosystem in cervical carcinogenesis and identified potential biomarkers for early detection.

## Introduction

Cervical cancer (CC) is primarily caused by the persistent infection of high-risk human papillomavirus (HPV) and ranks as the fourth most prevalent cancer in women [1]. There will be approximately 13,960 new cases of CC and 4310 related deaths in the United States during 2023 [2]. If diagnosed at an early stage and treated promptly, CC can be cured [1]. Throughout the past decades, CC incidence and mortality has declined in most parts of the world because of formalized cytology and HPV-based screening [3]. Genital HPV infection is prevalent, with approximately 85% of females infected at some point during their lives, but most HPV infections are transient and cleared spontaneously [4]. Previous studies showed the majority of low-grade squamous intraepithelial lesion (LSIL) and part of high-grade squamous intraepithelial lesion (HSIL) would regress within 2 years [5, 6]. Only a minority of LSIL progressed to CC [5]. Although co-testing with cytology and HPV is effective and recommended for screening, it is unpredictable whether an infection is transient, resulting in spontaneous regression of abnormalities, or persistent, leading to invasive cancer [7]. Therefore, new molecular diagnostics is warranted to make up for the deficiencies of existing screening methods.

Tumor microenvironment (TME), which consists of malignant cells, cancer-associated fibroblasts, and various immune cells (dendritic cells (DCs), B lymphocytes (B cells), T lymphocytes (T cells), monocytes, neutrophils, natural killer (NK) cells, macrophages, etc.), innately modulates tumor progression [8, 9]. According to the genetic and epidemiological evidence, HPV can alter the microenvironment to produce a protumorigenic state of immune suppression and evasion, ultimately contributing to the formation and progression of tumors [10]. Wang et al. [11] reported that the immune response between LSIL and HSIL was different. Particularly, immunosuppression and evasion occurred in HSIL, which contributed to tumorigenesis.

Single-cell RNA sequencing (scRNA-seq) can realize and identify complex cell populations and probe the molecular heterogeneity of TME at single-cell resolution, making it an ideal tool for cancer research [12, 13]. For instance, scRNA-seq has been conducted to investigate the structural heterogeneity of CC, as well as alterations in endothelial cells and fibroblasts [14–17]. At the same time, the heterogeneity of the tumor and its microenvironment has also been mapped and described throughout CC progression [18, 19]. Nevertheless, the possibility of detecting these alterations and incorporating them into screening still needs more investigations.

The detection of cervical exfoliated cells is recommended as the first step in the primary screening, by taking advantage of convenient, easily obtained, noninvasive, and easily accepted than cervical histopathological examinations. Thus, in this study, we examined cervical exfoliated cells using scRNA-seq to investigate heterogeneous and specific components of the TME in LSIL, HSIL, CC, as well as normal cervix (NC). Together, a comprehensive analysis of cervical exfoliated cells was carried out using scRNA-seq to identify potential biomarkers and molecular mechanisms that could serve as predictors for cervical neoplasia.

## Results

### scRNA-seq profiling of stepwise progression in CC

**Fig. 1:**
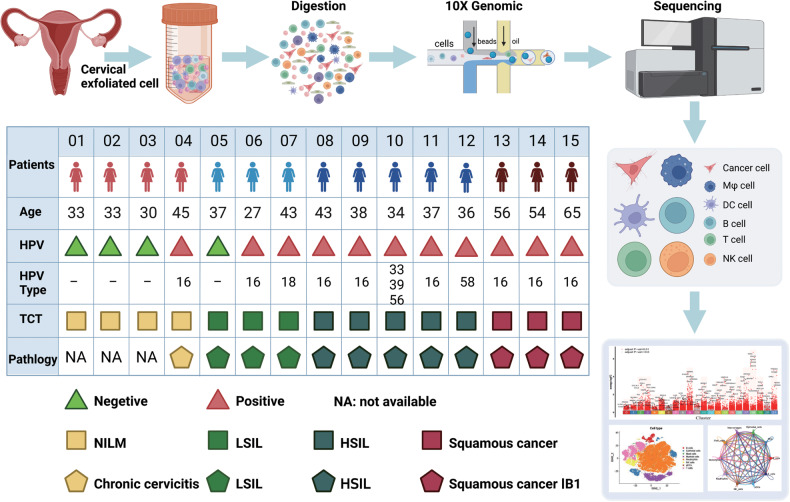
The schema of study design. Schematic showing the design of scRNA-seq experiment and patients information.

**Fig. 2:**
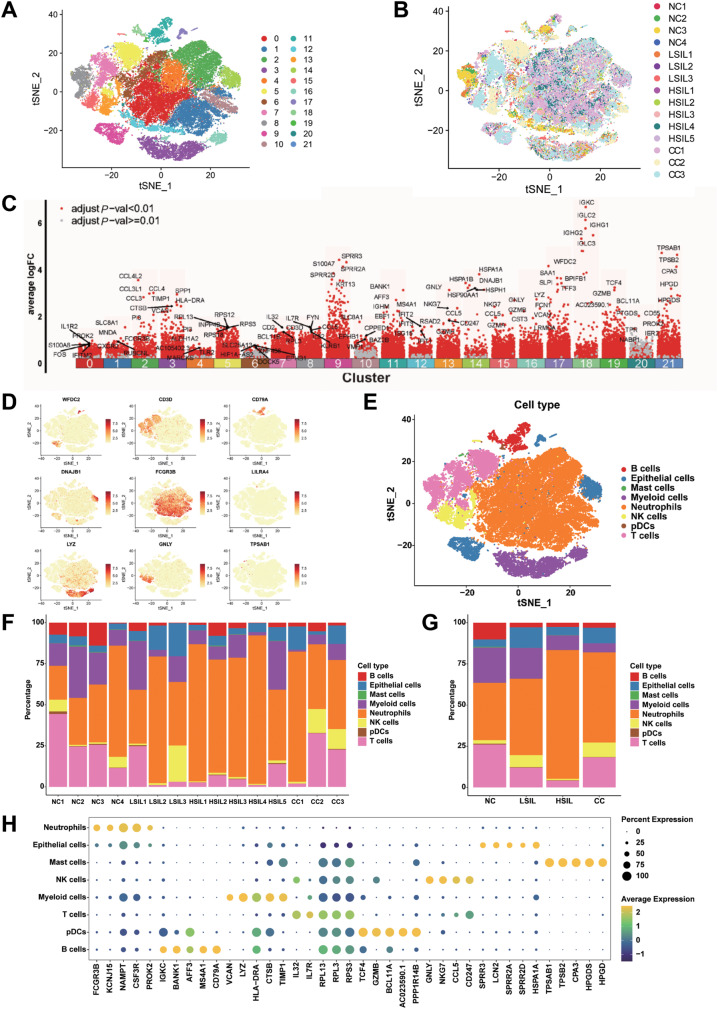
Single-cell atlas of cervical exfoliated cells in cervical carcinogenesis. **A** The t-distributed stochastic neighbor embedding (t-SNE) plots of 22 cellular clusters identified in 56,173 cells. Each dot represented a cell. **B** The t‐SNE plots of cells clustered by individual samples. **C** The top 5 genes were presented in each cluster. **D** The t‐SNE plots showed the expression and distribution of canonical cell markers. **E** The t‐SNE plot identified 8 cell types in cervical exfoliated cells. **F** Proportions of each cell type in each sample. **G** Average proportion of assigned cell types in different groups. **H** Bubble diagram showing the top 5 genes across distinct cell types. The X‐axis indicated the top genes of each cell subgroup and the Y‐axis indicated distinct cell subtypes.

To reveal the changing characteristics of TME during cervical carcinogenesis, we obtained scRNA-seq profiles from cervical exfoliated cells, which included NC (*n* = 4), LSIL (*n* = 3), HSIL (*n* = 5), and CC (*n* = 3) (Fig. 1). After quality control and filtering, a total of 56,173 cells were clustered into 22 clusters and visualized by graph-based t-distributed stochastic neighbor embedding (t-SNE) (Fig. 2A and Fig. S1). Of these, 8443, 3671, 18,879, and 25,180 cells were from NC, LSIL, HSIL, and CC, respectively. Moreover, distinct clustering was shown based on the per sample, which indicated the heterogeneity of individuals (Fig. 2B), and the top five upregulated genes of each cluster were displayed in Fig. 2C. Eight major cell populations were identified by their expression of known lineage markers with epithelial cells (*WFDC2*, *DNAJB1*, and *KRT17*), B cells (*CD79A*), T cells (*CD3D*), NK cells (*GNLY*), myeloid cells (*LYZ*), plasmacytoid dendritic cells (*LILRA4*), mast cells (*TPSAB1*), and neutrophils (*FCGR3B*) (Fig. 2D, E and Fig. S2).

The proportion of each cell type in the 15 samples was shown in Fig. 2F. The relative abundance of the eight major cell populations varied greatly by group (Fig. 2G). In general, the percentage of B cells decreased in LSIL, HSIL, and CC groups compared with NC group, while the percentage of neutrophils increased. Interestingly, the abundance of T cells generally reduced in the LSIL and HSIL groups compared with the NC group, which was partly rescued in CC group. In addition, the top five upregulated genes in each cell type were shown in Fig. 2H. These results demonstrated that significant immune infiltration was observed in the cervical exfoliated cells during cervical carcinogenesis, which was consistent with fresh tissue samples [18].

### Characterization of single-cell expression profiles for epithelial cells across different cervical lesions

**Fig. 3:**
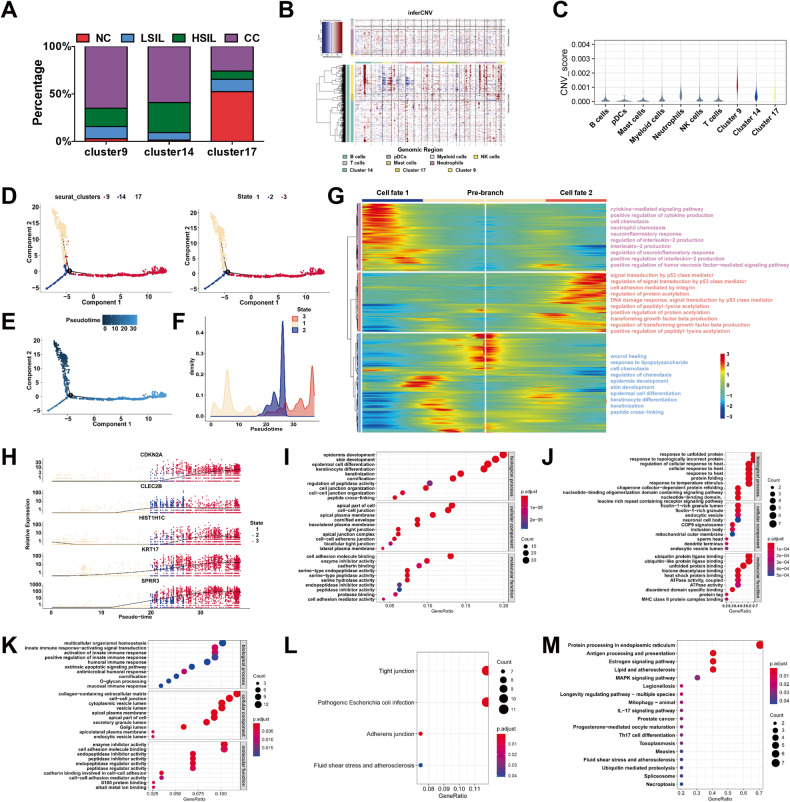
Identification and functional characterization of epithelial cells. **A** Bar graph showing the cell proportion of NC, LSIL, HSIL, and CC samples among cluster 9, 14, and 17. **B** Heatmap showing large-scale CNVs in single cells referenced to non-epithelial cell clusters. **C** Violin plots displayed the CNV score of each epithelial cell cluster. **D**, **E** Monocle 2 trajectory analysis of the epithelial cells annotated by cell subgroups (left panel), cell state (right panel) (**D**), and pseudotime (**E**) in cluster 9, cluster 14, and cluster 17. **F** Density distribution of epithelial cells along the pseudotime trajectory. **G** Heatmap depicting differentially expressed genes in epithelial cells based on the pseudotime trajectory. Color key from blue to red indicates relative expression levels from low to high. **H** The expression dynamics of representative genes are differentially expressed across pseudotime. All single cells in the three subclusters are colored based on (**D** and **E**) and ordered based on pseudotime. **I**–**K** The GO Analysis of cluster 9, cluster 14, and cluster 17. **L**, **M** The KEGG Analysis of cluster 9 and cluster 14.

Then, we analyzed epithelial cells (cluster 9, 14, and 17) in four groups (NC, LSIL, HSIL, and CC) to reveal the changing characteristics across different cervical lesions. In cluster 9 and 14, most cells were obtained from CC and HSIL groups, while the number of cells from NC and LSIL was scarce. However, the proportion of cells from NC was higher in cluster 17 compared with other clusters (Fig. 3A). To further confirm the properties of cells in three clusters, the copy number variations (CNVs) of epithelial cells were calculated (Fig. 3B). We measured the relative CNV score of each epithelial cluster compared to the non-epithelial cells. The results showed that cluster 9 had a highest variable of CNVs among the three epithelial clusters (Fig. 3C). Next, an analysis of pseudotime trajectory was conducted using Monocle 2 and each epithelial cell was ordered along trajectories based on its expression and transition characteristics. As shown in Fig. 3D, we revealed one branch of epithelial cell subtypes and indicated that epithelial cells exhibited three differentiation states during their development. To further clarify the relationship between cell states and subclusters, we performed a linear model analysis. The analysis of variance showed a significant difference of the variable state existed among the different seurat clusters (*P* < 0.05) (Fig. S3A). While Fig. 3E, F depicted the chronological order of cell subtypes differentiation, with darker-colored cells gradually transitioning into lighter-colored cells. It suggested that cluster 17 cells were located at the beginning and then differentiated into cluster 14 cells and cluster 9 cells. Subsequently, using the BEAM function, we presented the temporal gene expression of the distinct branch through a heatmap. By conducting Gene Ontology (GO) enrichment analysis to explore biological process, the results showed that one branch was mainly associated with signal transduction by p53 class mediator, regulation of signal transduction by p53 class mediator, DNA damage response, and signal transduction by p53 class mediator, which represented the triggering of p53 pathways, and the other was associated with immune cells-related signaling pathways, such as cytokine-mediated signaling pathway and positive regulation of cytokine production (Fig. 3G). Importantly, the expression of malignant cell markers (*KRT17*, *CLEC2B*, *HIST1H1C*, and *SPRR3*) and proliferation marker (*CDKN2A*) progressively increased with the alteration of cell states (Fig. 3H). Next, we employed GO and Kyoto Encyclopedia of Genes and Genomes (KEGG) pathway to discover the function under high expressed genes in those three clusters. Top 30 GO enrichments were shown. The genes specifically expressed in cluster 9 were largely involved with epidermis development and cell–cell junction (Fig. 3I). By comparison, genes expressed in cluster 14 were largely related to unfolded protein response and ATPase activity (Fig. 3J). In cluster 17, a great deal of enriched GO terms were activation of innate immune response and enzyme inhibitor activity (Fig. 3K). The KEGG enrichment analyses showed the upregulated genes in cluster 9 cells were significantly enriched in adherens junction, and tight junction (Fig. 3L), while those in cluster 14 were associated with IL-17 and MAPK pathway (Fig. 3M). According to our analysis and previous study [15], cancer cells in early phase had relatively high differentiation potential, and then the cells proliferated rapidly. During the late stages of CC growth, tumor cells demonstrated epidermis development and cell–cell junction. These results reflected the heterogeneity and complexity of CC.

### Characterization of epithelial cells subclusters during different cervical lesions

**Fig. 4:**
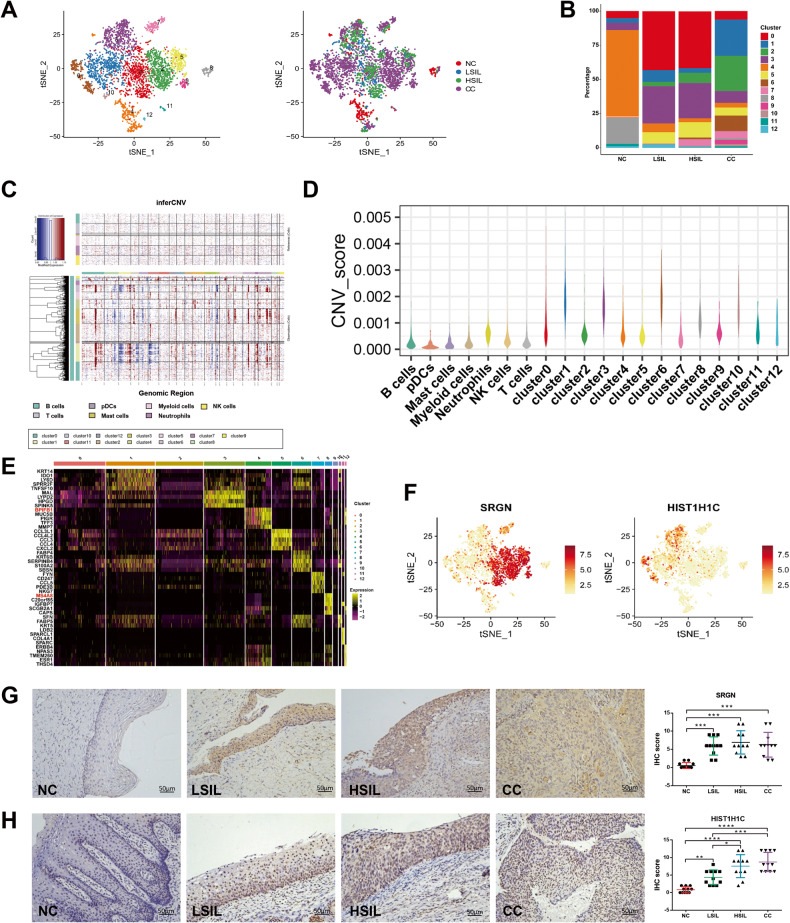
scRNA-seq profiles 13 subgroups of the epithelial cell cluster. **A** Reclustering of epithelial cells, color-coded by clusters (left) or group origin (right). **B** Average proportion of 13 subgroups of epithelial cell among NC, LSIL, HSIL, and CC samples. **C** Heatmap showing large-scale CNVs in single cells of epithelial cells referenced to non-epithelial cell clusters. **D** Violin plots displayed the CNV score of each epithelial cell subclusters. **E** Heatmap showing the differentially expressed genes (rows) of the epithelial cells across cluster (columns), with top genes indicated. **F** t-SNE plots showing the expression and distribution of *SRGN* and *HIST1H1C* in the subcluster of epithelial cells. **G**, **H** SRGN protein and HIST1H1C protein expression in NC, LSIL, HSIL, and CC tissues by immunohistochemistry. *P < 0.05, **P < 0.01, ***P < 0.001, ****P < 0.0001.

In order to completely understand the heterogeneity of malignant-derived cells, a total of 4,152 cervical epithelial cells were subclustered by t-SNE into 13 groups (Fig. 4A). Malignant cell identification: First, subcluster 1/2/3/6/10 almost exclusively presented in the LSIL, HSIL, and CC groups, while subcluster 4 and 8 took up the majority of NC group cells (Fig. 4B). Second, during the large-scale chromosomal CNV, subcluster 1/3/6/10 exhibited higher variable of CNV score compared with non-epithelial cells (Fig. 4C, D). Third, some malignant cell markers (*DSG3*, *FABP4*, *SFN*, *FABP5*, and *KRT5*) were detected [20–24]. The expression of *SFN*, *FABP5*, and *KRT5* were highly expressed in subclusters 1, 6, and 10. *DSG3* was highly expressed in subcluster 1 and 6, while *FABP4* was highly expressed in subcluster 6 (Fig. S3B). Based on the above evidences, we inferred cells from subclusters 1, 6, and 10 as malignant cells. Meanwhile, the analysis of pseudotime trajectory indicated that epithelial cells transitioned from non-malignant cells to malignant cells (Fig. S4A). At the same time, the cells from subcluster 4 and 8 exhibited high expression of antioncogenes, such as *BPIFB1* and *MS4A8* (Fig. 4E) [25, 26]. Meanwhile, the consensus nonnegative matrix factorization (cNMF) method was used to decompose mixed expression profiles of single cells into a linear combination of biologically interpretable gene expression programs (GEPs). 16 GEPs were deconvolved, which further clustered into four consensus modules (Fig. S4B). Intriguingly, we identified several novel genes including *SRGN* and *HIST1H1C* (Fig. 4F). The immunohistochemistry (IHC) staining of the SRGN and HIST1H1C in NC, LSIL, HSIL, and CC tissues was further detected. IHC scores in our study showed that the expression of SRGN protein was dramatically upregulated not only in CC but also in HSIL and LSIL compared with NC, while there was no difference among HSIL, LSIL, and CC (Fig. 4G). The expression of HIST1H1C protein was stepwise upregulated from NC to CC group, while there was no difference between HSIL and CC (Fig. 4H). To further evaluate the role of *SRGN* and *HIST1H1C* during the carcinogenesis of CC, the stable SRGN and HIST1H1C-overexpressed CC cells (SiHa and C33A) were established by infecting with lentivirals containing SRGN-pCDH or HIST1H1C-pCDH plasmid, respectively. The efficacy of SRGN overexpression was verified, which showed that SRGN expression was elevated by infecting with SRGN cDNA compared to the negative control (Fig. S5A). The CCK-8 assay displayed that SRGN overexpression strongly increased cell viability in both SiHa and C33A cells (Fig. S5B). Similarly, colony-forming assay was also conducted to assess the function of SRGN on cell proliferation, and it turned out that SRGN-overexpressed cells formed significantly more colonies (Fig. S5C, D). To further validate the pro-tumor function of SRGN in vivo, SiHa cells with overexpression of SRGN and its respective empty vectors were hypodermically injected into nude mice. Consistently, the tumor volume and weight remarkably increased in the SRGN overexpression groups compared to its control group, respectively (Fig. S5E–G). Meanwhile, the HIST1H1C overexpression was also confirmed using western blot. The transfected cells expressed a high expression of HIST1H1C (Fig. S6A). Colony formation and cell viability were enhanced by HIST1H1C overexpression in both SiHa and C33A cells (Fig. S6B–D). The tumor volume and weight also increased in the HIST1H1C overexpression groups compared to its control group (Fig. S6E–G). These characteristics indicated SRGN and HIST1H1C facilitated tumor growth and contributed to CC progression.

### Characterization of B cells in different cervical lesions

**Fig. 5:**
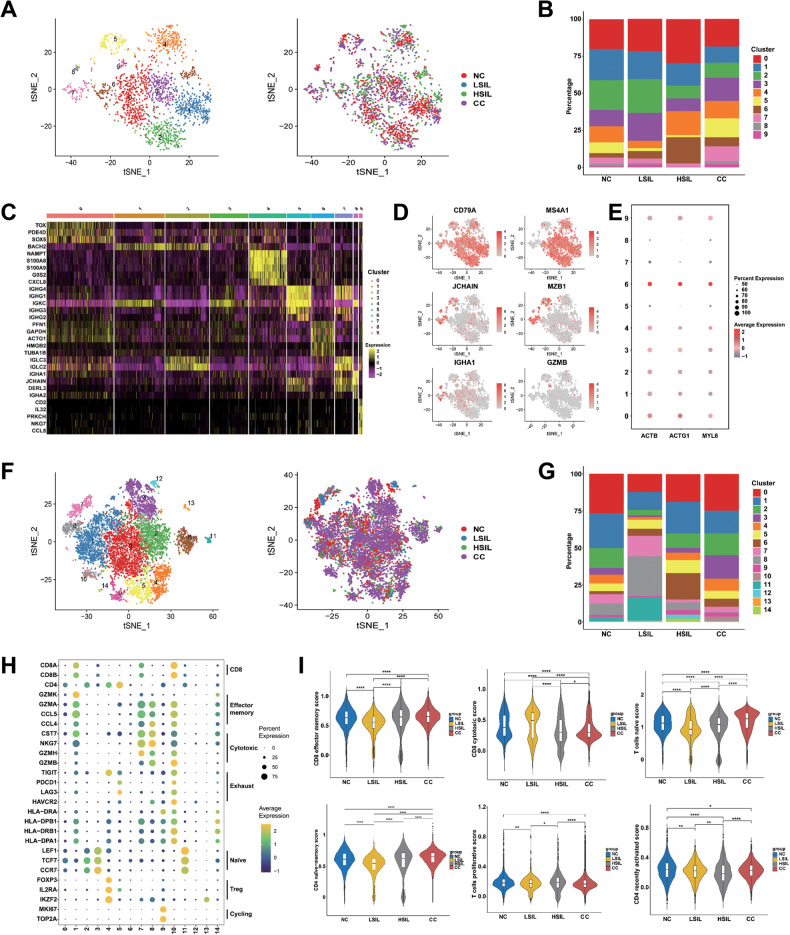
scRNA-seq profiles subgroups of the B cell and T cell cluster. **A** Reclustering of B cells, color-coded by clusters (left) or group origin (right). **B** Average proportion of 10 subgroups of the B cell among NC, LSIL, HSIL, and CC samples. **C** Heatmap showing the differentially expressed genes (rows) of B cells across cluster (columns), with top genes indicated. **D** The t‐SNE plots of the B cell biomarkers. **E** Expression of cell motility-related genes (*ACTB*, *ACTG1*, and *MYL6*) in the subcluster of B cells. The dot size is proportional to the fraction of marker-expressing cells in the group. **F** Reclustering of T cells, color-coded by clusters (left) or group origin (right). **G** Average proportion of 15 subgroups of the T cell of NC, LSIL, HSIL, and CC samples. **H** Expression of T cell-specific markers across different clusters. The dot size is proportional to the fraction of marker-expressing cells in the group. **I** Violin plots showing the scores of functional modules for four groups, using the AddModuleScore function. *P < 0.05, **P < 0.01, ****P < 0.0001.

The tumor-infiltrating B cells play a crucial role in modulating the development of tumors [27]. Nevertheless, little research has been conducted regarding the alteration of B cells in cervical carcinogenesis. B cells were selected and divided into 10 subgroups (Fig. 5A, B). Three major types of B cells were identified, including follicular B cells (subcluster 0, 1, 2, 3, 4, and 6, marked by *MS4A1* and *CD79A*), plasma B cells (subcluster 5, 7, and 8, marked by *JCHAIN*, *MZB1*, and *IGHA1*), and granzyme B‐secreting B cells (subcluster 9, marked by *GZMB*) (Fig. 5C, D). Among them, follicular B cells were predominant among all groups. There was a higher percentage of follicular B cells in LSIL and HSIL compared to the other groups, whereas the CC group showed the lowest percentage. A subcluster of follicular B cells, subcluster 6, was more abundant in the HSIL group and showed high expression of cell motility-related genes (*ACTB*, *ACTG1*, and *MYL6)* (Fig. 5E). Plasma B cells were enriched in CC group and expressed high levels of immunoglobulins genes (such as *IGHG4*, *IGHG3*, *IGHG2*, and *IGHG1* etc.) (Fig. 5B, C). Granzyme B‐secreting B cells could secrete granzyme B and were enriched in LSIL and CC groups (Fig. 5B). This suggested that B cells, especially plasma B cells and granzyme B‐secreting B cells might be stimulated to infiltrate in the CC and play a pivotal role in CC immunity.

### Distinct subpopulations of T cells during different cervical lesions

As T cells played an important role in tumor immunity, we subclustered them into 15 subclusters based on their characterizations (Fig. 5F, G). Particularly, cells from subcluster 1, 7, and 10 exhibited high levels of *CD8A* or *CD8B*, defining them as CD8^+^T cells. Subcluster 0, 2, 3, 4, 5, 9, 11, and 14 showed high levels of *CD4*, defining them as CD4^+^T cells (Fig. 5H).

Next, the subclusters with specific characteristics were analyzed. As for CD8^+^T cells, subcluster 1 was characterized as effector memory CD8^+^T cells due to specific expression of *GZMK*, *GZMA*, *CST7*, and a variety of cytokines-related genes (*CCL5* and *CCL4*). As shown in Fig. 5G–I (signature genes listed in Table S1), the percentage of effector memory CD8^+^T cells mainly decreased and had a lower effector memory score in LSIL group than HSIL and CC, suggesting that the protective effector memory CD8^+^T cell immunity was not established in LSIL. Agreeing with the previous study, our results showed the percentage of those cells increased in HSIL, suggesting an establishment of protective T cell immunity [18]. It was a long process to differ CD8^+^T cells from a naïve state to memory CD8^+^T cells. Subcluster 7 exhibited a high level of cytotoxic genes (*CST7*, *NKG7*, *GZMH*, and *GZMB*), commonly associated with cytotoxic T cells. In the LSIL group, these cells made up the highest percentage, whereas in the HSIL and CC groups, they comprised a lower percentage. The CD8 cytotoxic score in the LSIL group increased than HSIL group, and the score in the HSIL and CC groups decreased compared with NC group (Fig. 5G–I). Subcluster 10 was defined as exhausted CD8^+^T cells with abundant expression of exhaustion marker genes (*TIGIT*, *PDCD1*, *LAG3*, and *HAVCR2*) and antigen presentation genes (*HLA-DRA*, *HLA-DRB1*, *HLA-DPB1*, and *HLA-DPA1*). They were mainly enriched in CC group (Fig. 5G, H). Based on this evidence, LSIL presented an activated immune state, whereas tumor displayed an immunosuppressive state.

When it came to CD4^+^T cells, cells from subcluster 3 and 11 were considered as naïve T cells, characterized by expression of *LEF1*, *TCF7*, and *CCR7*. NC and LSIL groups showed a high percentage of subcluster 11, while HSIL and CC groups had a lower percentage. In contrast, subcluster 3 extremely increased in CC groups. T cells naïve scores decreased in both LSIL and HSIL groups, while they increased in CC groups (Fig. 5G–I). Subcluster 4 was defined as regulatory T cells with expression of *FOXP3*, *IL2RA*, and *IKZF2*. They were enriched in CC group and also showed an exhaustion state (Fig. 5G, H). Based on its high expression of *MKI67* and *TOP2A*, subcluster 9 was classified as cycling T cells. They were slightly increased in HSIL and CC groups (Fig. 5G, H). Additionally, subcluster 8 also expressed effector marker genes (*NKG7*, *CST7*, and *GZMH*), but not *CD8*, indicating that it was a natural killer T cell. Its distribution was extremely increased in LSIL groups (Fig. 5G, H). According to above results, the immune microenvironment gradually shifted toward immunosuppression during cervical carcinogenesis.

### Distinct subpopulations of NK cells during different cervical lesions

**Fig. 6:**
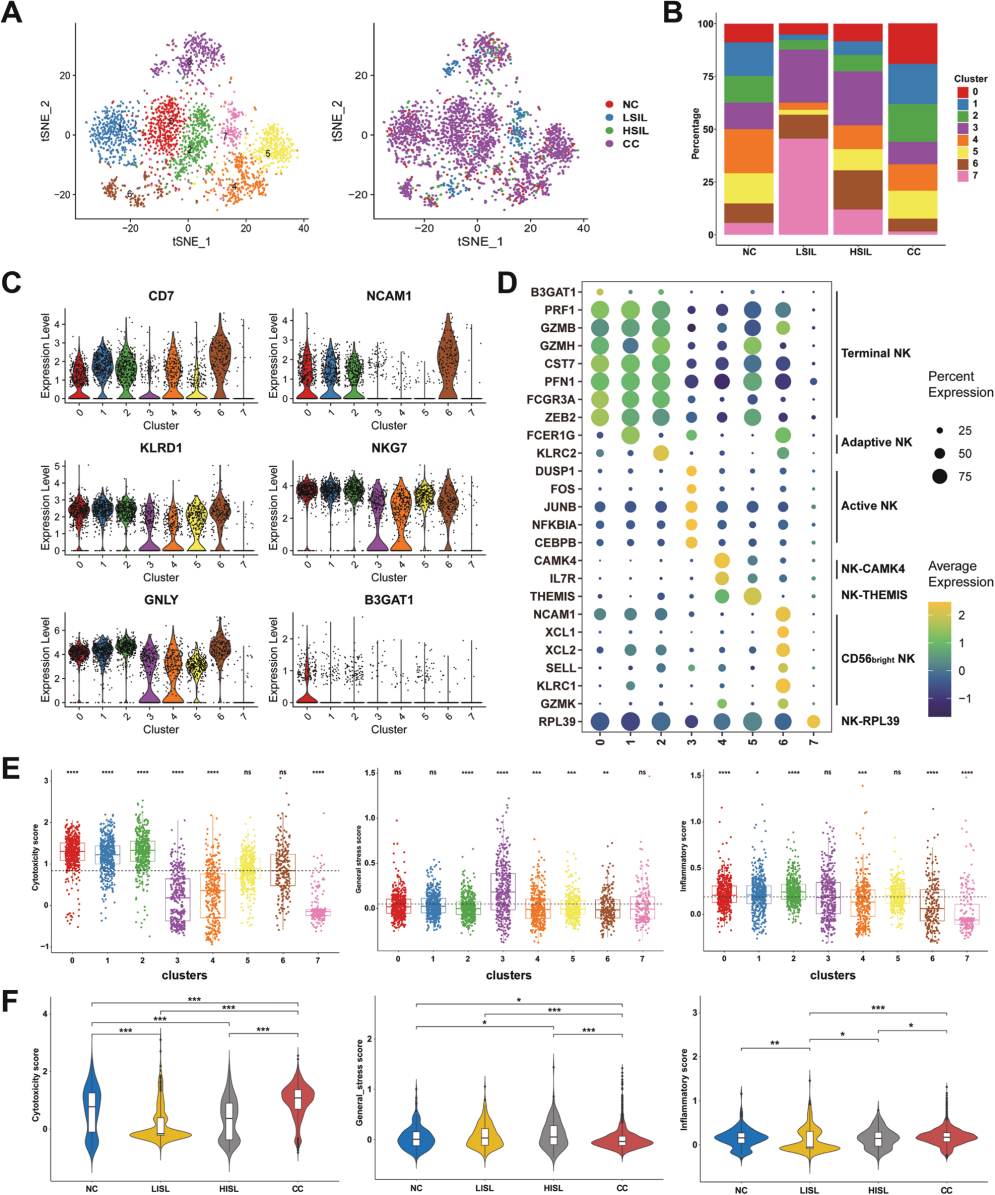
scRNA-seq profiles 8 subgroups of the NK cell cluster. **A** Reclustering of NK cells, color-coded by clusters (left) or group origin (right). **B** Average proportion of 8 subgroups of the NK cell among NC, LSIL, HSIL, and CC samples. **C** Violin plots displayed the expression of NK cell-specific markers across different clusters. **D** Expression of NK cell-specific markers across different clusters. The dot size is proportional to the fraction of marker-expressing cells in the group. **E** The expression pattern of functional genes in NK cells. **F** Violin plots showing the scores of functional modules for four groups, using the AddModuleScore function. *P < 0.05, **P < 0.01, ***P < 0.001, ****P < 0.0001, ns: not significant.

The NK cell is an innate lymphoid cell that has antimicrobial and anti-tumor properties [28]. Additionally, NK cells were further divided into 8 subsets (Fig. 6A, B). Most of the cells had a high level of well-defined NK-cell markers, such as *CD7*, *NCAM1*, *KLRD1*, *NKG7*, *GNLY*, and *B3GAT1* (Fig. 6C) [29]. Cells from subcluster 6 characterized by high expression of *NCAM1* (*CD56*) were identified as CD56^bright^ NK cells. A higher percentage of CD56^bright^ NK cells was observed in the HSIL and LSIL groups than those in NC and CC, while those cells expressed high levels of resting NK cells markers, such as *XCL1* and *KLRC1* [30]. Cells from subcluster 0 and 1 have high level of *PRF1*, *GZMB*, *GZMH*, *CST7*, *PFN1*, and *FCGR3A*, which were identified as terminal NK cells. Meanwhile, subcluster 0 cells displayed relatively high levels of *B3GAT1* and *ZEB2*, which had previously been identified as mature terminal NK. Subcluster 2 represented the adaptive NK cells and showed high expression of *KLRC2* and low expression of *FCER1G*. Cells from subcluster 3 were considered as active NK cells, characterized by expression of *DUSP1*, *FOS*, *JUNB*, *NFKBIA*, and *CEBPB*. Subcluster 4, 5, and 7 were defined as NK-CAMK4, NK-THEMIS, and NK-RPL39 with special high expression of *CAMK4*, *THEMIS*, and *RPL39*, respectively (Fig. 6D). Next, the function of NK cells was assessed using AddModuleScore function (signature genes listed in Table S2). Terminal and adaptive NK cells exhibited higher cytotoxicity score compared with that in other subclusters. Active NK cells had a weak cytotoxicity and a high stress score. The inflammatory score was low in CD56^bright^ NK and NK-RPL39. Compared with the NC group, the cytotoxicity score decreased in LSIL and HSIL groups and rescued in the CC group. In contrast, NK cells in HSIL group exhibited a higher stress score and NK cells in CC group had a lower stress score compared with the NC group (Fig. 6E, F). Taken together, the CD56^bright^ NK cells were in a resting state and NK cells in LSIL had a low inflammatory score, which might contribute to accelerate the progression of cervical lesions, but the role of NK cells in CC was complex and unclear, which needed further detailed experimental studies.

### Characterization of myeloid cells during different cervical lesions

**Fig. 7:**
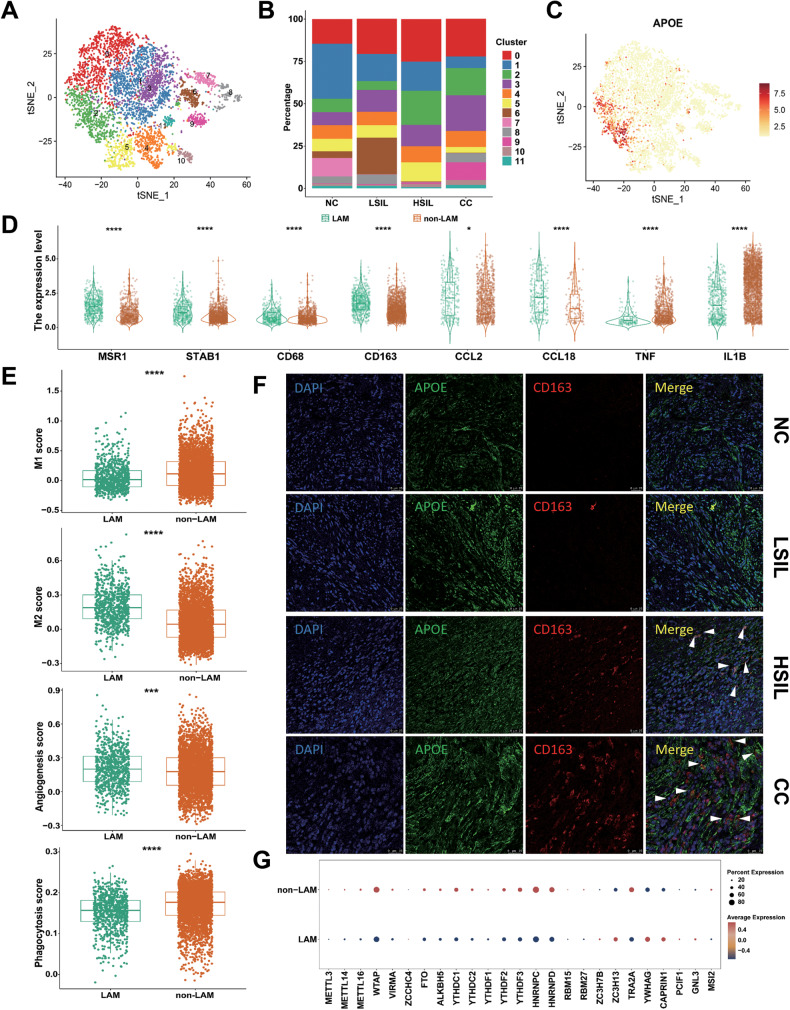
Functional states of myeloid cells in the NC, LSIL, HSIL, and CC groups. **A** The t‐SNE projection of myeloid cells demonstrating 12 main subclusters. **B** Average proportion of 12 subgroups of the myeloid cells among NC, LSIL, HSIL, and CC samples. **C** The t‐SNE plots showing the expression and distribution of *APOE* among myeloid cells. **D** Differences of M1/M2-like genes and cytokines between LAM and non-LAM. **E** Comparison of M1, M2, phagocytosis, and angiogenesis score between LAM and non-LAM. **F** Representative images for multiplexed immunofluorescence staining of LAM in NC, LSIL, HSIL, and CC tissues (CD163, red; APOE, green). DAPI was used to highlight all nucleus. **G** The expression of m6A methylation associated RNA transcripts between LAM and non-LAM. *P < 0.05, ***P < 0.001, ****P < 0.0001.

In the myeloid cells, 12 subclusters were observed, including 2 subclusters (subcluster 6 and 7) of monocytes (*FCN1*), 3 subclusters (subcluster 4, 5, and 10) of DCs (*LAMP3*, *CD1C*, *FCER1A*, *XCR1*, and *CLEC9A*), and 7 subclusters (subcluster 0, 1, 2, 3, 8, 9, and 11) of macrophages (Figs. 7A and S7A).

Subcluster 7 was special appearance in NC group, and they faded in LSIL, HSIL, and CC groups. On the contrary, subcluster 9 gradually increased and accounted for a significant percentage of CC group (Fig. 7B). Notably, the functional analysis revealed that subcluster 7 was enriched in pathways related to virus response, such as defense response, response to virus, and viral process (Fig. S7B, C). As shown in Fig. S7D, E, the subcluster 9 was associated with the histone deacetylase activity, which involved in the initiation and development of cancer [31]. These alters might accelerate the cervical carcinoma initiation.

Specifically, compared with LSIL group, cells in HSIL and CC groups showed a relatively higher percentage of subcluster 2, which expressed high expression of marker genes, such as *APOE*, *APOC1*, *GPNMB*, *SPP1*, and *CD163* (Fig. 7B, C, Fig. S7A, S7F). We defined this subcluster as lipid-associated macrophages (LAMs) according to the LAMs signatures from previous studies [32–34]. The remaining macrophages were classified as non-LAMs.

Macrophages are usually classified into pro-inflammatory (M1-like) or anti-inflammatory (M2-like) phenotypes. In the present study, genes and cytokines which could differ between M1 and M2-like macrophages were detected in LAMs and non-LAMs. We found the expression of M2-like signatures (such as *MSR1*, *STAB1*, *CD68*, *CD163*, *CCL2*, and *CCL18*) was upregulated in LAMs. Meanwhile, the non-LAMs expressed high levels of M1-like signatures (*TNF* and *IL1B*) (Fig. 7D). Next, the functional phenotypes of LAMs and non-LAMs were assessed using AddModuleScore function (signature genes listed in Table S3). Notably, LAMs exhibited a higher M2 score, while non-LAMs expressed a higher canonical M1 score. As expected, LAMs exhibited significantly lower phagocytosis scores, while exhibited remarkably higher angiogenesis scores. Compared to LAMs, non-LAMs showed notably higher phagocytosis scores, but significantly lower angiogenesis scores (Fig. 7E). Collectively, it could be postulated that LAMs displayed a tumor-supporting phenotype similar to M2-like macrophages. They increased the ability of angiogenesis and decreased the ability of phagocytosis. To further confirm the presence of LAMs, the H&E and multiplexed immunofluorescence staining were performed in NC, LSIL, HSIL, and CC tissues. The H&E staining of NC, LSIL, HSIL, and CC tissues was showed in Fig. S7G. The marker genes of *APOE* and *CD163* were chosen to label the LAMs. As shown in Fig. 7F and Fig. S7H, the number of *APOE*^+^/*CD163*^+^ macrophages was specifically enriched in CC group, less abundant in HSIL group, and almost absent in LSIL and NC groups. To further explore the role of APOE in macrophage, the stable APOE overexpression in human monocyte THP-1 cells were established by infecting with lentiviruses APOE overexpression and control of APOE overexpression. The infection efficiency of lentivirus was confirmed. The results of PCR and Western blot analysis suggested that the mRNA and protein level of APOE were upregulated in transfected THP-1 cells (Fig. S8A, B). Then, THP-1 cells were treated with phorbol-12-myristate-13-acetate (PMA) to generate M0 macrophages. CCK-8 assay suggested that the conditioned medium (CM) from APOE overexpression macrophages could promote the activity of SiHa (Fig. S8C) and Caski (Fig. S8D) cells compared with NC group. In contrast, the CM from APOE overexpression macrophages did not alter the activity of cervical epithelial cell (ECT) compared with NC group (Fig. S8E). Besides, transwell experiments also showed that the CM from APOE overexpression macrophages accelerated the migration of SiHa (Fig. S8F) and Caski (Fig. S8G) cells. Besides, the CM from APOE overexpression macrophages did not alter the migration of ECT cells compared with NC group (Fig. S8H). These results further confirmed that LAMs might be able to promote CC progression.

Recent studies indicated that the alteration of N6-methyladenosine (m6A) in macrophage could alter its tumor immunogenicity and anti-tumor activity [35, 36]. To determine whether m6A modifications intrinsically affect macrophages, the expression of m6A modification marker genes in macrophages were examined. In macrophages, no stable changes were observed in the expression of m6A regulators among the NC, LSIL, HSIL, and CC groups (Fig. S9A). Intriguingly, compared to non-LAMs, the majority of m6A regulators (*WTAP*, *HNRNPC*, *HNRNPD*, etc.) were lowly expressed in LAMs (Fig. 7G). It indicated that the changes of m6A modification in LAMs might have an impact on its role in tumor immune microenvironment.

Next, we found that the DC subclusters were divided into two categories, conventional DC1 (cDC1) and conventional DC2 (cDC2). Cells from subcluster 4 and 5 expressed the classical cDC2 signatures (*CD1C* and *FCER1A*). Additionally, they expressed antigen-presenting genes, including *HLA-DPB1*, *HLA-DQA1*, *HLA-DPA1*, and *HLA-DQB1* (Fig. S7A). The main function of these cells was to acquire tumor antigen and primed the T cells to recognize it. Subcluster 4 cells also expressed high levels of *LAMP3*, indicating mature DCs. The mature DCs (subcluster 4) had also high expression levels of *BIRC3*, *FSCN1*, *CD86*, *CD80*, and *CCL22* representing an activated state (Fig. S7A). The percentage of cDC2 cells increased in HSIL group and decreased in CC group (Fig. 7B). Compared with cDC2 cells, subcluster 10 cells expressed higher level of marker genes of cDC1 (*XCR1* and *CLEC9A*). In addition, it was found that a greater percentage of cDC1 cells were found in the HSIL and CC groups compared to the other groups (Fig. 7B). Those cells had a high expression level of *IDO1* (Fig. S7A), which inhibited T cell activation and induced T cell differentiation into suppressive regulatory T cells [37]. In summary, these above results revealed the distinct lineages and states of DC cells in the TME of CC.

### Cell–cell communication networks in NC, LSIL, HSIL, and CC

**Fig. 8:**
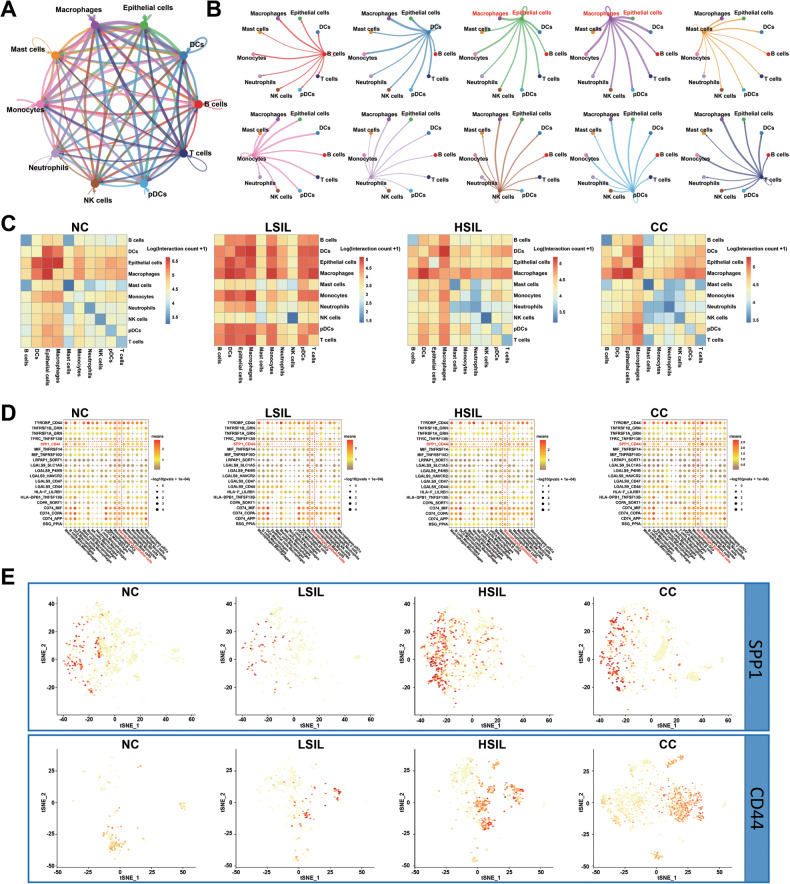
Cell–cell communication among all cell types in the cervical exfoliated cells microenvironment. **A** Circos plot showing the potential cell interactions among ten major cell types by CellphoneDB. Node size indicates interactions; edge width represents the number of significant ligand-receptor pairs. **B** Detailed view of the ligands expressed by each major cell types and the cells expressing the cognate receptors primed to receive the signal. **C** Heatmap depicting the significant ligand-receptor cellular interactions in different groups (The color bar represents the interaction counts between two different types of cells). **D** Dot plot indicating the ligand-receptor pairs between different cells. **E** t‐SNE plots showing the expression and distribution of *SPP1* in the subcluster of macrophages, and *CD44* in the subcluster of epithelial cells.

Based on the expression of ligand-receptor gene in single cells, cell–cell interactions were investigated to show the intercellular interactions in the cervical microenvironment (Fig. 8A). The broad range of molecular interactions were further demonstrated by the identification of broadcast ligand-receptor pairs among major cell types (Fig. 8B). Additionally, a heatmap plot function was used to analyze the interaction of the ligand and receptor among the major cell types (Fig. 8C). It was indicated that macrophages demonstrated extensive communications with epithelial cells. In order to understand the communications between macrophages and epithelial cells, the receptor-ligand interactions were further studied. Previous studies had found that SPP1 involved in tumor progression by interacting with CD44 [38, 39]. In our results, a communication through *SPP1*-*CD44* interaction between macrophages and epithelial cells in cervical exfoliated cells was also identified. The interaction was rarely detected in NC and LSIL groups but relatively higher expressed in the HSIL and CC groups (Fig. 8D), suggesting that *SPP1*-*CD44* axis might act a critical role in cervical carcinogenesis. The level of *SPP1* in macrophages was upregulated in HSIL and CC groups compared to NC and LSIL groups (Fig. S9B). The level of *CD44* in epithelial cells was upregulated in HSIL group compared to NC group. Meanwhile, compared to LSIL, the level of *CD44* in epithelial cells was both upregulated in HSIL and CC groups (Fig. S9C). According to our results, macrophages and epithelial cells might interact through *SPP1*-*CD44* axis, which might contribute to the cervical carcinogenesis.

**Fig. 9:**
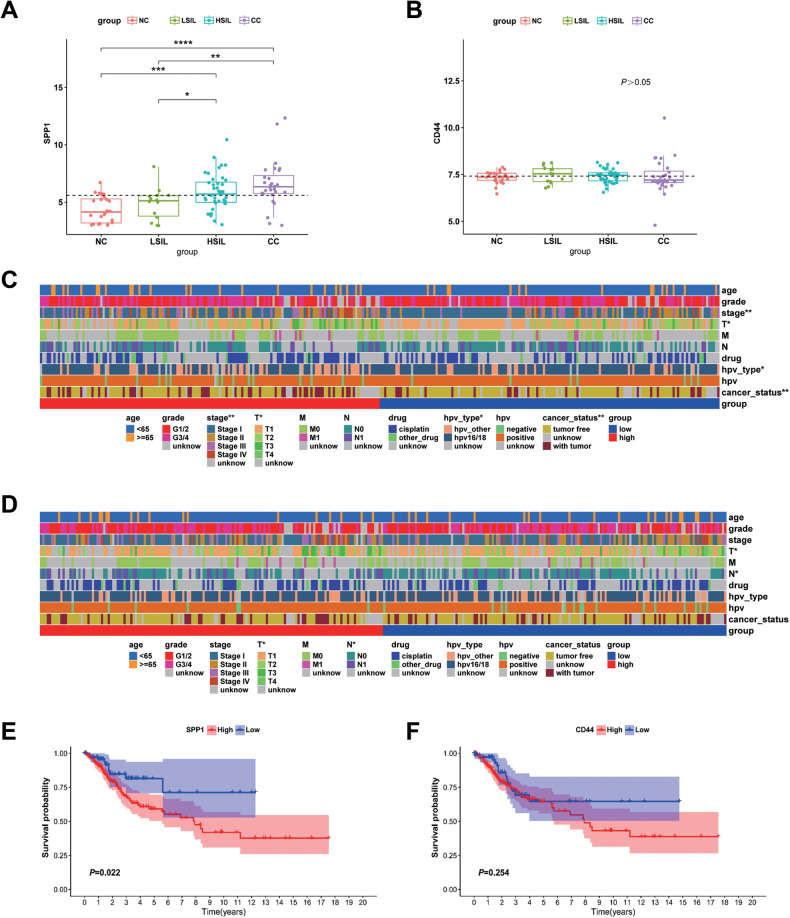
Determination of the expression and prognostic role of *SPP1* and *CD44* in CC. **A**, **B** the expression of *SPP1* and *CD44* in NC, LSIL, HSIL, and CC from GEO database, respectively. **C**, **D** Heatmap visualizing clinical features, as well as *SPP1* and *CD44* expression in the TCGA-CESC dataset. **E**, **F** Kaplan–Meier survival curve of *SPP1* and *CD44* in CC from TCGA-CESC database. *P < 0.05, **P < 0.01, ***P < 0.001, ****P < 0.0001.

To verify the pro-tumorigenic effects of *SPP1*-*CD44* axis, the expression levels of *SPP1* and *CD44* were detected using GSE63514 dataset. The *SPP1* expression was significantly higher in HSIL and CC than that in NC, which was similar with our results, but *CD44* expression was not related to the formation of CC (Fig. 9A, B). Moreover, Chi-square test identified tumor stage, HPV type, and tumor size were positively correlated with *SPP1* expression, while tumor size and numbers of positive nodes were related to *CD44* expression in CC patients from The Cancer Genome Atlas cervical squamous cell carcinoma (TCGA-CESC) (Fig. 9C, D and Table S4). Furthermore, the survival data from TCGA-CESC dataset was also analyzed to explore the correlation of *SPP1* and *CD44* expression with prognosis of CC. Not surprisingly, an increase in *CD44* expression had no effect on prognosis in CC, whereas an increase of *SPP1* expression predicted a poor overall survival in CC (Fig. 9E, F). Overall, these findings confirm that *SPP1* is highly expressed in the macrophages and interacts with epithelial cells through *SPP1*-*CD44* axis. The activation of *SPP1*-*CD44* axis plays a significant role in tumorigenesis, and is significantly associated with poor prognosis in CC.

## Discussion

Herein, we establish a single-cell transcriptional atlas of cervical exfoliated cells from 15 samples ranging from NC to CC with different histologic subtypes. This atlas has comprehensively described the characteristics of TME, malignant cells, and variations in cell–cell crosstalk networks during tumor progression. The data provides a deeper insight for improving cervical precancer diagnosis, refining patient stratification, as well as offering valuable resources for future studies to discover biomarkers and potential therapeutic targets.

According to previous studies, CC tissues and adjacent normal tissues were chosen for scRNA-seq analysis to reveal the alterations of fibroblasts and endothelial cells as well as the heterogeneity in malignant cells [14–17]. Meanwhile, by performing scRNA-seq of normal cervical tissue, precancerous lesions, CC, and metastatic lymph nodes, the heterogeneity of malignant cells and TME in CC was mapped [18, 19]. In summary, previous studies in CC focused more on samples from tissues to reveal the heterogeneity by using scRNA-seq. In this study, for the first time, cervical exfoliated cells were collected for scRNA-seq analysis to explore the biomarkers for early detection of CC.

In agreement with the previous study, the evolution of exfoliated epithelial cells was stepwise during cervical carcinogenesis [19]. According to scRNA-seq analyses, we found cervical exfoliated cells were composed of epithelial cells, neutrophils, mast cells, and immune cells. Meanwhile, we identified several novel genes including *SRGN* and *HIST1H1C*. *SRGN* was recently reported as an oncogene to promote the metastasis of esophageal and nasopharyngeal carcinomas [40, 41]. HIST1H1C was upregulated and promoted hepatocarcinogenesis [42]. However, the function of *SRGN* and *HIST1H1C* has not been explored in cervical cancer. Interestingly, it could be inferred from our investigation that *SRGN* and *HIST1H1C* might be novel biomarkers that contribute to CC development.

Tumor microenvironments contain various immune cells, which are crucial to tumorigenesis and tumor progression. Previous studies have described the alterations of TME across the CC progression through scRNA-seq [17–19]. Meanwhile, the immune microenvironment of LSIL remains undescribed. Through scRNA-seq of cervical exfoliated cells from LSIL, our study identified several distinct immune cells subpopulations and described the alterations of microenvironment of LSIL. Our research revealed that exfoliated cells from LSIL composed a large part of cytotoxic T cells and natural killer T cells. Additionally, the LSIL group presented a higher CD8 cytotoxic score compared with HSIL and tumor groups. In summary, our study showed that LSIL displayed an activated immune state, in contrast to HSIL and tumor.

Macrophages are one of the essential components of the TME and show a complex heterogeneity [43]. Accumulated evidence revealed that LAMs shared a high expression of typical genes (such as *APOE*, *APOC1*, *GPNMB*, *TREM2*, *SPP1* etc.) and played a central role in tumor immunity [32, 34]. A clinical cohort indicated that LAMs were enriched in clear cell renal carcinoma patients with recurrences and could serve as a prognostic biomarker as well as a candidate therapeutic target [44]. Additionally, an *APOE*^+^/*C1QB*^+^ macrophage was identified in intrahepatic cholangiocarcinoma, which could reshape the chronic inflammation and predict a poor prognosis [45]. In addition, a previous scRNA-seq reported that *APOE*^+^ macrophages were enriched in advanced CC and associated with a poor survival [19]. These results confirmed that *APOE*^+^ macrophages existed in CC and played a role in tumor development. The *APOE*^+^ macrophages were found to be similar to LAMs in our study. Accordingly, our study confirmed LAMs could be detected in the cervical exfoliated cells. At the same time, it was indicated that LAMs played multiple roles in tumorigenesis. Since LAMs exhibited preferential genes expression in angiogenesis, those type of macrophages were likely to possess anti-inflammatory properties similar to M2-like macrophages. We then performed multiplexed immunofluorescence staining to further confirm the enrichment of LAMs in HSIL and CC tissues. There was a significant amount of LAMs in both the HSIL and CC groups, which contradicted a previous finding that *APOE*^+^ macrophages were just specifically increased in the CC group [19]. It is highly possible that the difference is due to the fact that we used cervical exfoliated cells instead of tissue specimens for scRNA-seq analysis. Moreover, individual variations may also contribute to these differences. Therefore, LAMs may act as a protumor factor to accelerate oncogenic events and could serve as a biomarker for predicting progression of LSIL into HSIL.

m6A is the most prevalent and reversible mRNA modification, which regulates tumor cell in various biological processes [46]. Recently, several studies indicated that m6A could also modulate the function of tumor-associated macrophages (TAMs) and alter its anti-tumor responses in TME [35]. Yin et al. [36] identified *METTL3* depletion in macrophages promoted orchestrates cancer progression. When activated by lipopolysaccharide in vitro, *METTL3*-deficient macrophages could not produce the enough tumor necrosis factor (TNF)-α and contribute to tumor growth [41]. By scRNA-seq, Dong et al. [47] demonstrated the deficiency of *METTL14* in macrophages inhibited the anti-tumor function of CD8^+^ T cells and promoted tumor growth. In our study, one of the most striking findings was that the majority of the m6A modification marker genes were low expressed in LAMs. It was possible that the modification of the m6A in LAMs altered their function. However, it remains to be elucidated how m6A methylation affects LAMs to regulate the formation of CC.

Macrophage is an important component of myeloid cells [48]. It was identified that *SPP1* acted as an oncogene involved in carcinogenesis and CC progression, meanwhile its upregulation decreased the sensitivity of cisplatin and predicted a poor prognosis for HPV positive CC patients [49–51]. In a recent study, CC patients could be clearly divided into two subclusters according to the *C1QC*^+^ and *SPP1*^+^ TAMs gene signatures. Compared with *C1QC*^high^ and *SPP1*^low^ TAMs, patients with *SPP1*^high^ and *C1QC*^low^ TAMs had a worse prognosis and lower level of immune cell infiltration [52]. Additionally, it had been demonstrated that *SPP1*/*CD44* interaction mediated crosstalk between macrophages and glioma cells. As a result, patients with high level of both *SPP1* and *CD44* had an increased macrophages infiltration and a poor prognosis in glioma [53]. At the same time, a similar conclusion was found in hepatocellular carcinoma (HCC), reporting that macrophages interacted with HCC malignant cells through the *SPP1*-*CD44* axis. High expression of *SPP1* and *CD44* indicated a worse prognosis of HCC [54]. In the present study, the CellPhoneDB had identified multiple ligand-receptor interactions. Furthermore, an interaction was observed between macrophages and malignant cells through the *SPP1-CD44* axis. According to our findings, the *SPP1-CD44* interaction expressed a low level in NC and LSIL, but highly in HSIL as well as CC, suggesting that macrophages derived *SPP1* served as an imperative component in the formation of CC. Combining *SPP1* and *CD44* can serve as an early biomarker for cervical cancer diagnosis and prognosis.

More than 70% LSIL will regress and 10% of LSIL will progress to HSIL [6]. In light of this, it is necessary to identify the specific microenvironment and differences of LSIL in order to determine the key factors affecting tumorigenesis. To our best knowledge, there is still no relevant scRNA-seq study about it. To perform scRNA-seq on LSIL, we selected patients with LSIL on cervical cytology and collected the samples before the performance of colposcopy and biopsy. Additionally, the diagnosis was further confirmed by pathology. In the present study, novel diagnostic biomarkers for CC based on bioinformatics analysis were established by verifying clinical samples from our center, which will have a tremendous clinical importance.

Nevertheless, there are still some drawbacks to the present study. We notice that a few of subcluster 1 of epithelial cells are presented in NC group, though the percentage of these cells is low (3%). Agreeing with the previous study, it may be a false positive result [55]. Our results indicated that neutrophils were the predominant cell type in some samples. Although, we had excluded the patients suffering the acute vulvovaginal candidiasis and bacterial vaginosis. Our finding supported the previous reports claiming neutrophils acted as the dominant immune cell type in the cervix [56–58]. The percentage of neutrophils was different with samples collected from biopsies [59]. At the same time, the tumor-associated neutrophils would drive the progress of non-small cell lung cancer had been revealed by scRNA-seq [60]. Hence, neutrophils were still included for scRNA-seq in order to avoid missing information on the relationship between neutrophils and CC. Unfortunately, we did not find a clear connection between them. On the other hand, further validation of our findings, including the molecular characteristics of cells during cervical cancer development, is required.

## Materials and methods

### Patients and sample collection

During July 2021 to October 2021, patients with normal and abnormal cervical cytology were enrolled at the Second Affiliated Hospital of Wenzhou Medical University, whose samples of cervical exfoliated cells were collected for scRNA-seq. Patients with abnormal cytology including LSIL, HSIL, and squamous cell carcinoma were referred to colposcopy and biopsy in this study. The pathological results were diagnosed by two senior pathologists, and the histological results were classified as LSIL, HSIL, and squamous cancer. Exclusion criteria included: (1) participants with the history of chemotherapy or radiotherapy; (2) acute vulvovaginal candidiasis, bacterial vaginosis, gonorrhea, or mycoplasma infections; (3) immunosuppression due to organ transplantation or human immunodeficiency virus; (4) current pregnancy or lactation; and (5) previous treatment for cervical lesions.

### Isolation of single cells

The suspension of exfoliated cell was filtered, centrifuged, resuspended, and then incubated with red blood cell lysis buffer. After incubation, the dead cells among suspension were removed using a Miltenyi^®^Dead Cell Removal Kit. The pellet of cells was resuspended in PBS (containing 0.04% bovine serum albumin) and adjusted to 1000–1200 cells per liter. Trypan blue exclusion was used to determine cell viability.

### 10 × Genomics library and sequencing

According to the manufacturer’s instructions previously described [61], single-cell suspensions were loaded into 10 × Chromium to capture single cells. Afterward, the following cDNA amplification and library construction were conducted by LC-Bio Technology. Library sequencing was performed on the Illumina NovaSeq sequencing system.

### Quality control and cell-type identification

For quality control, Seurat (version 4.3.0) was used to count unique molecular identifiers (UMIs) and mitochondrial genes. Cells with more than 100 UMIs and less than 25% mitochondrion-derived UMI counts were selected. This study selected the top 20 components and first 2000 variable genes. The “ScaleData” function was used to regress the inflow of UMIs and the percentage of mitochondrion-derived UMI counts. Subsequently, the main cell clusters were identified by Seurat’s “FindClusters” function. Unbiased cell type recognition was visualized by t-SNE [62]. Cell types were annotated based on their canonical marker genes expression via “SingleR” function and manually checked genes from the CellMarker database and published papers [63]. The method of cNMF was used to discover gene expression programs in tumor samples [64].

### Pseudotime trajectory analysis

The trajectory analysis was utilized by Monocle 2 package (version 2.26.0) to reveal the cell-state transitions [65]. The “DDRTree” function was applied to reduce the dimensions with default settings. The trajectory was visualized by “plot_cell_trajectory” function.

### Copy number alteration inference

The CNV was evaluated by “infercnv” R package (version 1.12.0) to differentiate between malignant and non-malignant cells [66]. The different genomic locations of CNVs were presented.

### Differently expressed genes (DEGs) identification and function annotation

The DEGs between subcluster 7 and subcluster 9 from myeloid cells were calculated via “FindMarkers” in Seurat package with default parameters. GO and KEGG analysis of marker genes and DEGs were performed using cluster Profiler R-package. According to GO terms, it included three categories: molecular function, cellular component, and biological process.

### Calculation of functional module scores

The functional module scores were calculated to evaluate the functions of specific cell clusters, using “AddModuleScore” function in Seurat. The related gene lists for T cell, NK cell, and macrophage were presented respectively in Tables S1, S2, and S3, respectively.

### Cell culture

THP-1, ECT, CC cell lines (SiHa, Caski, and C33A) and 293T cells were obtained from Type Culture Collection of China Center and incubated at 5% CO_2_ at 37 °C. All cells were cultured in DMEM or 1640 medium (BI, USA) supplemented with 10% fetal bovine serum (FBS, GIBCO, USA) and authenticated by short tandem repeat.

### Virus production, cell transfection, and macrophage generation

The Youbio Biosciences Inc provided the SRGN and HIST1H1C cDNA expression vector. By transfecting lentivectors with psPAX2 and pMD2.G, lentivector particles were produced in 293T. The lentiviruses APOE overexpression and control were obtained from Shanghai GeneChem Co., Ltd. According to the manufacturer’s protocol, cells were transfected. Puromycin was added to select the infected cells after the virus was produced and infected the target cells. The efficacy was verified by western blot after transfection. THP-1 cells were pretreated with PMA for 24 h to generate M0 macrophages. Then, M0 macrophages were cultured with 1640 medium containing 10% FBS. After 48 h, the CM was collected for further analysis.

### Western blot

Western blot experiments were performed as previously [67]. Anti-SRGN (1:100, sc-374657, Santa Cruz Biotechnology), Anti-HIST1H1C (1:1000, ab4086, abcam), Anti-APOE (1:1000, #13366, Cell Signaling Technology), anti-Tubulin (1:5000, #T0023, Affinity Biosciences) and anti-β-Actin (1:4000, #T0022, Affinity Biosciences) were used as primary antibodies. The blots were imaged with the ChemiScope System (Clinx, China).

### Colony formation assay and CCK-8

Colony formation assays were performed with SiHa (300 cells/well) and C33A (1000 cells/well) cells being seeded into 6-well plate and cultured in standard cell media until cell colonies were formed. After washed by PBS, the colonies were fixed in paraformaldehyde and then stained with crystal violet for visualization. The colonies with more than 50 cells were analyzed. Cells were seeded into 96-well plate with 10% FBS media or CM, and then incubated for indicated time. After each well had been incubated at 37 °C for 2 h with 10 μl of CCK-8 reagent (Meilunbio, China), the optical density (OD) of each well was determined using a microplate reader.

### Tumor xenograft experiment

Female BALB/C nude mice (4-week old) were purchased by the Animal Experiment Center of Wenzhou Medical University and randomly divided into four groups. SiHa cells (3 × 10^6^ stably transfected with SRGN overexpression, HIST1H1C overexpression, their corresponding control vector) were subcutaneously injected into the flank of mice. Tumor sizes were calculated by the formula: Volume = width^2^ × length/2. This assay was approved by the Experimental Animal Center of Wenzhou Medical University (wydw2023-0459).

### Migration assay

The migration assay was performed as previously [68]. The CM from macrophages were added into the bottom chambers. SiHa, Caski, and ECT cells were seeded in upper chambers with serum-free medium and co-culture with CM for 24 h. Then, the upper chambers underwent the fixing process and staining. The cells were photographed and counted.

### H&E, IHC, and multiplexed immunofluorescence staining

Tissue slices including NC, LSIL, HSIL, and CC were obtained from the Second Affiliated Hospital of Wenzhou Medical University. After underwent deparaffinization, the tissue sections were performed H&E staining. For IHC, the tissue sections underwent deparaffinization and subsequent antigen retrieval via microwave heating with sodium citrate. Subsequently, the sections were stained with rabbit anti-human SRGN (ABclonal Cat# A6951, RRID:AB_2767509) or rabbit anti-human HIST1H1C (Proteintech Cat# 19649-1-AP, RRID:AB_10694432) at 4 °C for 12 h. On the following day, goat anti-rabbit secondary antibody (ZSGB-BIO, China) was used. The sections were then subjected to hematoxylin counter-staining and 3,3’-Diaminobenzidine staining. For multiplexed immunofluorescence staining, the 5 μm tissue sections were deparaffinized and antigen retrieval as above, and then stained with rabbit anti-human CD163 (Proteintech Cat# 16646-1-AP, RRID:AB_2756528) and mouse anti-human APOE (Proteintech Cat# 66830-1-Ig, RRID:AB_2882173) together at 4 °C for 12 h. Subsequently, the tissues were stained with anti-mouse Alexa488 (1:250, Invitrogen) and anti-rabbit Alexa594 (1:250, Invitrogen) conjugated secondary antibodies for 1 h. After that, DAPI was used to stain nucleus. The slides were imaged using a microscope.

### Online data processing

GSE63514 was utilized to obtain expression profile of 24 normal cervical epithelium, 14 LSIL, 40 HSIL, and 28 cervical squamous epithelial cancer [69]. In addition, the expression levels and clinical information of 304 patients with cervical cancer were downloaded from TCGA-CESC database.

### Cell–cell communication analysis

The CellPhoneDB (version 4.0.0) software was used to analyze intercellular communications between different cell types from single-cell transcriptome data. All counts had been logarithmically transformed, quantile normalized, and performed as the previous report [18]. The ligands and receptors expressed in more than 10% of the cells in the specific cluster were chosen and analyzed. Then, pairwise comparisons between all cell types were performed, and the false-positive interaction was filtered. Only interactions of ligand-receptor with *P* < 0.05 were considered significant.

### Statistical analysis

The normality of data was tested. Continuous variables were presented as means and SDs if normally distributed and medians and IQRs if not. An unpaired Student’s *t*-test or Wilcoxon test was conducted using R as appropriate. *P*-values were adjusted for multiple testing, and *P*-values < 0.05 were considered statistically significant.

### Supplementary information

supplementary material

Table S1

Table S2

Table S3

Table S4

Table S5

Full and unccropped western blots

aj-checklist

## Data Availability

The data supporting the findings of this study are available from the corresponding author on reasonable request.

## References

[1] Cohen PA, Jhingran A, Oaknin A, Denny L (2019). Cervical cancer. Lancet.

[2] Siegel RL, Miller KD, Wagle NS, Jemal A (2023). Cancer statistics, 2023. CA Cancer J Clin.

[3] Arbyn M, Weiderpass E, Bruni L, de Sanjose S, Saraiya M, Ferlay J (2020). Estimates of incidence and mortality of cervical cancer in 2018: a worldwide analysis. Lancet Glob Health.

[4] Chesson HW, Dunne EF, Hariri S, Markowitz LE (2014). The estimated lifetime probability of acquiring human papillomavirus in the United States. Sex Transm Dis.

[5] Palefsky JM, Lee JY, Jay N, Goldstone SE, Darragh TM, Dunlevy HA (2022). Treatment of anal high-grade squamous intraepithelial lesions to prevent anal cancer. N Engl J Med.

[6] Salvado A, Miralpeix E, Sole-Sedeno JM, Kanjou N, Lloveras B, Duran X (2021). Predictor factors for conservative management of cervical intraepithelial neoplasia grade 2: cytology and HPV genotyping. Gynecol Oncol.

[7] Sawaya GF, Huchko MJ (2017). Cervical cancer screening. Med Clin North Am.

[8] Hinshaw DC, Shevde LA (2019). The tumor microenvironment innately modulates cancer progression. Cancer Res.

[9] Mao X, Xu J, Wang W, Liang C, Hua J, Liu J (2021). Crosstalk between cancer-associated fibroblasts and immune cells in the tumor microenvironment: new findings and future perspectives. Mol Cancer.

[10] Yuan Y, Cai X, Shen F, Ma F (2021). HPV post-infection microenvironment and cervical cancer. Cancer Lett.

[11] Wang Y, He M, Zhang G, Cao K, Yang M, Zhang H (2021). The immune landscape during the tumorigenesis of cervical cancer. Cancer Med.

[12] Lei Y, Tang R, Xu J, Wang W, Zhang B, Liu J (2021). Applications of single-cell sequencing in cancer research: progress and perspectives. J Hematol Oncol.

[13] Ren X, Liang J, Zhang Y, Jiang N, Xu Y, Qiu M (2022). Single-cell transcriptomic analysis highlights origin and pathological process of human endometrioid endometrial carcinoma. Nat Commun.

[14] Gu M, He T, Yuan Y, Duan S, Li X, Shen C (2021). Single-cell RNA sequencing reveals multiple pathways and the tumor microenvironment could lead to chemotherapy resistance in cervical cancer. Front Oncol.

[15] Li C, Guo L, Li S, Hua K (2021). Single-cell transcriptomics reveals the landscape of intra-tumoral heterogeneity and transcriptional activities of ECs in CC. Mol Ther Nucleic Acids.

[16] Li C, Wu H, Guo L, Liu D, Yang S, Li S (2022). Single-cell transcriptomics reveals cellular heterogeneity and molecular stratification of cervical cancer. Commun Biol.

[17] Ou Z, Lin S, Qiu J, Ding W, Ren P, Chen D, (2022). Single-nucleus RNA sequencing and spatial transcriptomics reveal the immunological microenvironment of cervical squamous cell carcinoma. Adv Sci.

[18] Li C, Hua K (2022). Dissecting the single-cell transcriptome network of immune environment underlying cervical premalignant lesion, cervical cancer and metastatic lymph nodes. Front Immunol.

[19] Liu C, Zhang M, Yan X, Ni Y, Gong Y, Wang C (2023). Single-cell dissection of cellular and molecular features underlying human cervical squamous cell carcinoma initiation and progression. Sci Adv.

[20] Brown L, Waseem A, Cruz IN, Szary J, Gunic E, Mannan T (2014). Desmoglein 3 promotes cancer cell migration and invasion by regulating activator protein 1 and protein kinase C-dependent-Ezrin activation. Oncogene.

[21] Zhao SG, Chen WS, Das R, Chang SL, Tomlins SA, Chou J (2019). Clinical and genomic implications of luminal and basal subtypes across carcinomas. Clin Cancer Res.

[22] Li G, Wu Q, Gong L, Xu X, Cai J, Xu L (2021). FABP4 is an independent risk factor for lymph node metastasis and poor prognosis in patients with cervical cancer. Cancer Cell Int.

[23] Du N, Li D, Zhao W, Liu Y. Stratifin (SFN) regulates cervical cancer cell proliferation, apoptosis, and cytoskeletal remodeling and metastasis progression through LIMK2/cofilin signaling. Mol Biotechnol. 2023. 10.1007/s12033-023-00946-1. 10.1007/s12033-023-00946-1 PMC11549181 37946061

[24] George Warren W, Osborn M, Yates A, Wright K, O’Sullivan SE (2023). The emerging role of fatty acid binding protein 5 (FABP5) in cancers. Drug Discov Today.

[25] Wei F, Wu Y, Tang L, He Y, Shi L, Xiong F (2018). BPIFB1 (LPLUNC1) inhibits migration and invasion of nasopharyngeal carcinoma by interacting with VTN and VIM. Br J Cancer.

[26] Sun L, Zhang Y, Zhang C (2018). Distinct expression and prognostic value of MS4A in gastric cancer. Open Med.

[27] Engelhard V, Conejo-Garcia JR, Ahmed R, Nelson BH, Willard-Gallo K, Bruno TC (2021). B cells and cancer. Cancer Cell.

[28] Liu S, Galat V, Galat Y, Lee YKA, Wainwright D, Wu J (2021). NK cell-based cancer immunotherapy: from basic biology to clinical development. J Hematol Oncol.

[29] Yang C, Siebert JR, Burns R, Gerbec ZJ, Bonacci B, Rymaszewski A (2019). Heterogeneity of human bone marrow and blood natural killer cells defined by single-cell transcriptome. Nat Commun.

[30] Wang C, Yu Q, Song T, Wang Z, Song L, Yang Y (2022). The heterogeneous immune landscape between lung adenocarcinoma and squamous carcinoma revealed by single-cell RNA sequencing. Signal Transduct Target Ther.

[31] Shetty MG, Pai P, Deaver RE, Satyamoorthy K, Babitha KS (2021). Histone deacetylase 2 selective inhibitors: a versatile therapeutic strategy as next generation drug target in cancer therapy. Pharmacol Res.

[32] Timperi E, Gueguen P, Molgora M, Magagna I, Kieffer Y, Lopez-Lastra S (2022). Lipid-associated macrophages are induced by cancer-associated fibroblasts and mediate immune suppression in breast cancer. Cancer Res.

[33] Sun K, Xu R, Ma F, Yang N, Li Y, Sun X (2022). scRNA-seq of gastric tumor shows complex intercellular interaction with an alternative T cell exhaustion trajectory. Nat Commun.

[34] Jaitin DA, Adlung L, Thaiss CA, Weiner A, Li B, Descamps H (2019). Lipid-associated macrophages control metabolic homeostasis in a Trem2-dependent manner. Cell.

[35] Li X, Ma S, Deng Y, Yi P, Yu J (2022). Targeting the RNA m(6)A modification for cancer immunotherapy. Mol Cancer.

[36] Yin H, Zhang X, Yang P, Zhang X, Peng Y, Li D (2021). RNA m6A methylation orchestrates cancer growth and metastasis via macrophage reprogramming. Nat Commun.

[37] Xing X, Yang F, Huang Q, Guo H, Li J, Qiu M (2021). Decoding the multicellular ecosystem of lung adenocarcinoma manifested as pulmonary subsolid nodules by single-cell RNA sequencing. Sci Adv.

[38] Nallasamy P, Nimmakayala RK, Karmakar S, Leon F, Seshacharyulu P, Lakshmanan I (2021). Pancreatic tumor microenvironment factor promotes cancer stemness via SPP1-CD44 axis. Gastroenterology.

[39] Wei J, Marisetty A, Schrand B, Gabrusiewicz K, Hashimoto Y, Ott M (2019). Osteopontin mediates glioblastoma-associated macrophage infiltration and is a potential therapeutic target. J Clin Invest.

[40] Zhu Y, Lam AKY, Shum DKY, Cui D, Zhang J, Yan DD (2021). Significance of serglycin and its binding partners in autocrine promotion of metastasis in esophageal cancer. Theranostics.

[41] Wang YL, Ren D, Lu JL, Jiang H, Wei JZ, Lan J (2022). STAT3 regulates SRGN and promotes metastasis of nasopharyngeal carcinoma through the FoxO1-miR-148a-5p-CREB1 axis. Lab Invest.

[42] Wang Q, Chen Y, Xie Y, Yang D, Sun Y, Yuan Y (2022). Histone H1.2 promotes hepatocarcinogenesis by regulating signal transducer and activator of transcription 3 signaling. Cancer Sci.

[43] Lei X, Lei Y, Li JK, Du WX, Li RG, Yang J (2020). Immune cells within the tumor microenvironment: Biological functions and roles in cancer immunotherapy. Cancer Lett.

[44] Obradovic A, Chowdhury N, Haake SM, Ager C, Wang V, Vlahos L (2021). Single-cell protein activity analysis identifies recurrence-associated renal tumor macrophages. Cell.

[45] Bao X, Li Q, Chen J, Chen D, Ye C, Dai X (2022). Molecular subgroups of intrahepatic cholangiocarcinoma discovered by single-cell RNA sequencing-assisted multiomics analysis. Cancer Immunol Res.

[46] He L, Li H, Wu A, Peng Y, Shu G, Yin G (2019). Functions of N6-methyladenosine and its role in cancer. Mol Cancer.

[47] Dong L, Chen C, Zhang Y, Guo P, Wang Z, Li J (2021). The loss of RNA N(6)-adenosine methyltransferase Mettl14 in tumor-associated macrophages promotes CD8(+) T cell dysfunction and tumor growth. Cancer Cell.

[48] Varol C, Mildner A, Jung S (2015). Macrophages: development and tissue specialization. Annu Rev Immunol.

[49] Zhao K, Ma Z, Zhang W (2021). Comprehensive analysis to identify SPP1 as a prognostic biomarker in cervical cancer. Front Genet.

[50] Chen X, Xiong D, Ye L, Yang H, Mei S, Wu J (2019). SPP1 inhibition improves the cisplatin chemo-sensitivity of cervical cancer cell lines. Cancer Chemother Pharmacol.

[51] Deepti P, Pasha A, Kumbhakar DV, Doneti R, Heena SK, Bhanoth S (2022). Overexpression of Secreted Phosphoprotein 1 (SPP1) predicts poor survival in HPV positive cervical cancer. Gene.

[52] Li X, Zhang Q, Chen G, Luo D (2021). Multi-omics analysis showed the clinical value of gene signatures of C1QC(+) and SPP1(+) TAMs in cervical cancer. Front Immunol.

[53] He C, Sheng L, Pan D, Jiang S, Ding L, Ma X (2021). Single-cell transcriptomic analysis revealed a critical role of SPP1/CD44-mediated crosstalk between macrophages and cancer cells in glioma. Front Cell Dev Biol.

[54] Liu Y, Zhang L, Ju X, Wang S, Qie J (2022). Single-cell transcriptomic analysis reveals macrophage-tumor crosstalk in hepatocellular carcinoma. Front Immunol.

[55] Zhang T, Zhuang L, Muaibati M, Wang D, Abasi A, Tong Q (2023). Identification of cervical cancer stem cells using single-cell transcriptomes of normal cervix, cervical premalignant lesions, and cervical cancer. EBioMedicine.

[56] Mohammadi A, Bagherichimeh S, Perry MC, Fazel A, Tevlin E, Huibner S (2020). The impact of cervical cytobrush sampling on cervico-vaginal immune parameters and microbiota relevant to HIV susceptibility. Sci Rep.

[57] Hunter PJ, Sheikh S, David AL, Peebles DM, Klein N (2016). Cervical leukocytes and spontaneous preterm birth. J Reprod Immunol.

[58] Mohd Zaki A, Hadingham A, Flaviani F, Haque Y, Mi JD, Finucane D (2022). Neutrophils dominate the cervical immune cell population in pregnancy and their transcriptome correlates with the microbial vaginal environment. Front Microbiol.

[59] Trifonova RT, Lieberman J, van Baarle D (2014). Distribution of immune cells in the human cervix and implications for HIV transmission. Am J Reprod Immunol.

[60] Wu F, Fan J, He Y, Xiong A, Yu J, Li Y (2021). Single-cell profiling of tumor heterogeneity and the microenvironment in advanced non-small cell lung cancer. Nat Commun.

[61] Chen S, Cui W, Chi Z, Xiao Q, Hu T, Ye Q (2022). Tumor-associated macrophages are shaped by intratumoral high potassium via Kir2.1. Cell Metab.

[62] Aran D, Looney AP, Liu L, Wu E, Fong V, Hsu A (2019). Reference-based analysis of lung single-cell sequencing reveals a transitional profibrotic macrophage. Nat Immunol.

[63] Zhang X, Lan Y, Xu J, Quan F, Zhao E, Deng C (2019). CellMarker: a manually curated resource of cell markers in human and mouse. Nucleic Acids Res.

[64] Kotliar D, Veres A, Nagy MA, Tabrizi S, Hodis E, Melton DA (2019). Identifying gene expression programs of cell-type identity and cellular activity with single-cell RNA-Seq. Elife.

[65] Trapnell C, Cacchiarelli D, Grimsby J, Pokharel P, Li S, Morse M (2014). The dynamics and regulators of cell fate decisions are revealed by pseudotemporal ordering of single cells. Nat Biotechnol.

[66] Tirosh I, Venteicher AS, Hebert C, Escalante LE, Patel AP, Yizhak K (2016). Single-cell RNA-seq supports a developmental hierarchy in human oligodendroglioma. Nature.

[67] Lin M, Zhang J, Bouamar H, Wang Z, Sun LZ, Zhu X (2022). Fbxo22 promotes cervical cancer progression via targeting p57(Kip2) for ubiquitination and degradation. Cell Death Dis.

[68] Sheng B, Zhao B, Dong Y, Zhang J, Wu S, Ji H (2023). Copine 1 predicts poor clinical outcomes by promoting M2 macrophage activation in ovarian cancer. Carcinogenesis.

[69] den Boon JA, Pyeon D, Wang SS, Horswill M, Schiffman M, Sherman M (2015). Molecular transitions from papillomavirus infection to cervical precancer and cancer: Role of stromal estrogen receptor signaling. Proc Natl Acad Sci USA.

