# Supplementary material for: Single-cell RNA sequencing of cervical exfoliated cells reveals potential biomarkers and cellular pathogenesis in cervical carcinogenesis
Source: Cell Death Dis. 2024 Feb 12;15(2):130. doi: 10.1038/s41419-024-06522-y (PMC10861450; doi:10.1038/s41419-024-06522-y)
Supplement: Supplementary file 1 — supplementary material [file 41419_2024_6522_MOESM1_ESM.docx]

**
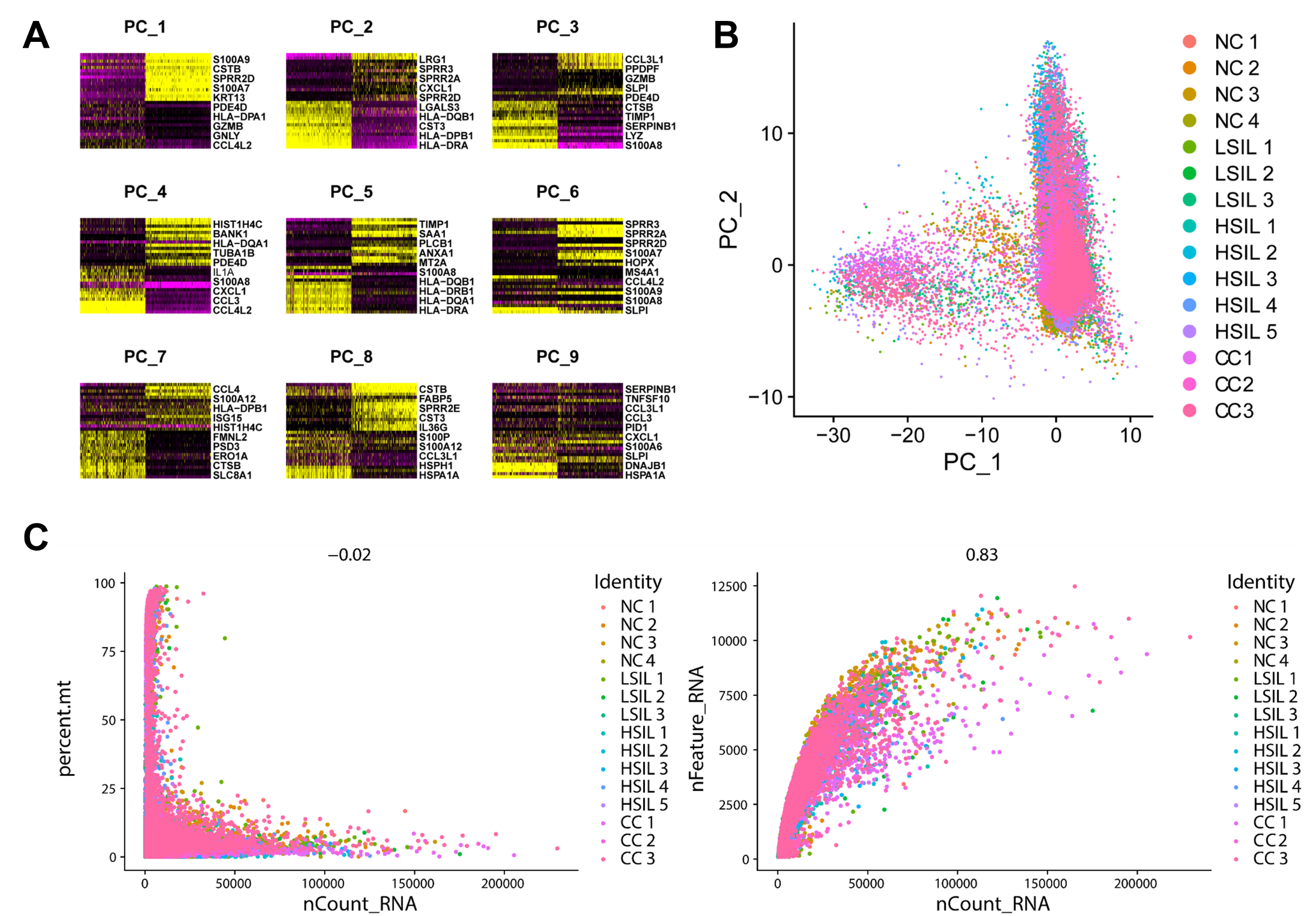
**

**Fig. S1. A** Heatmap of top 9 principal components. **B** PCA was used for dimensionality reduction color coded by sample. **C** Scatter plots showing the correlation between the percentage of mitochondrial genes and the mRNA reads, together with the relationship between sequencing depth and the number of detected genes.

**
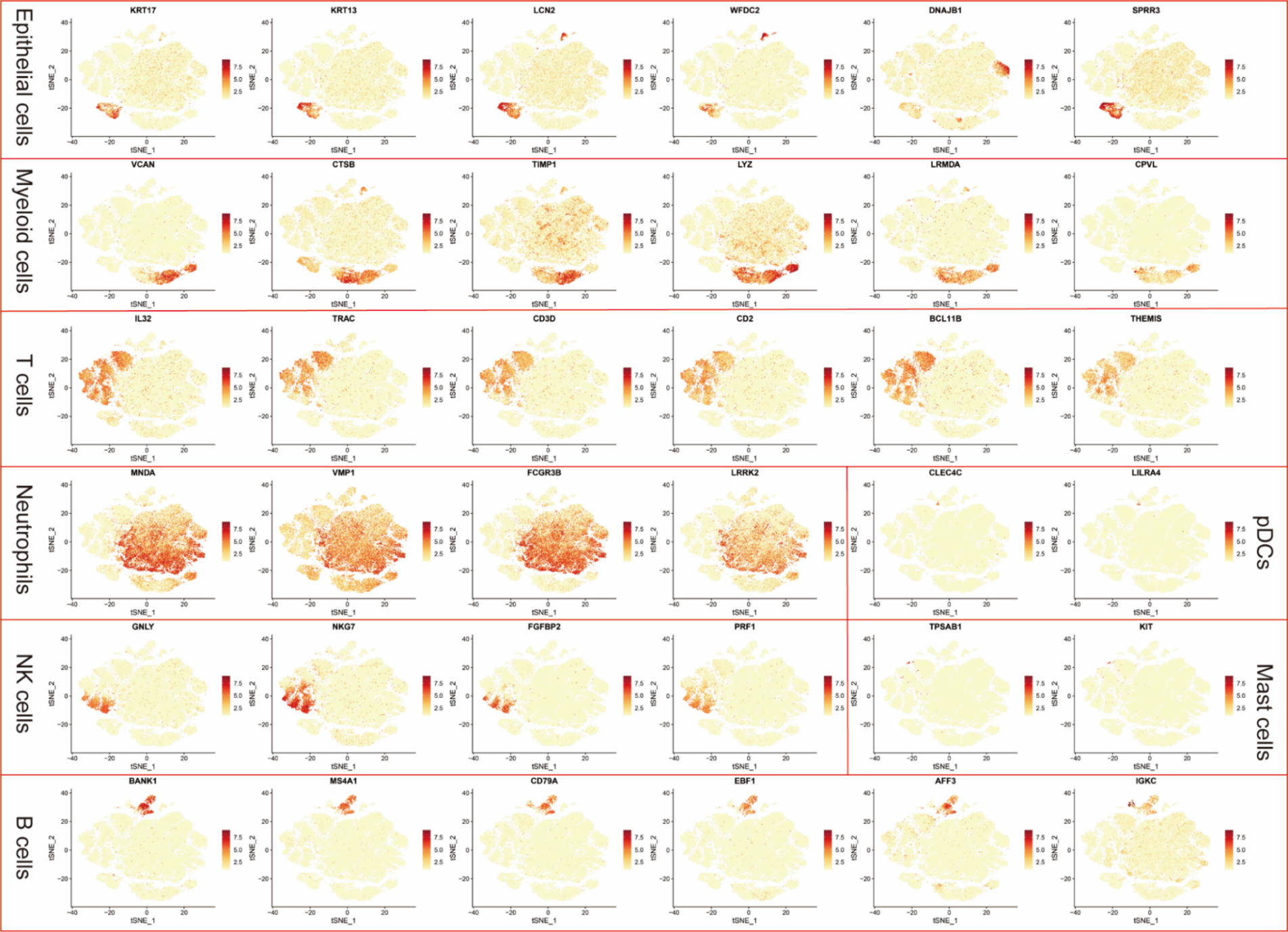
**

**Fig. S2.** The t‐SNE plots showing the expression and distribution of canonical cell markers.

**
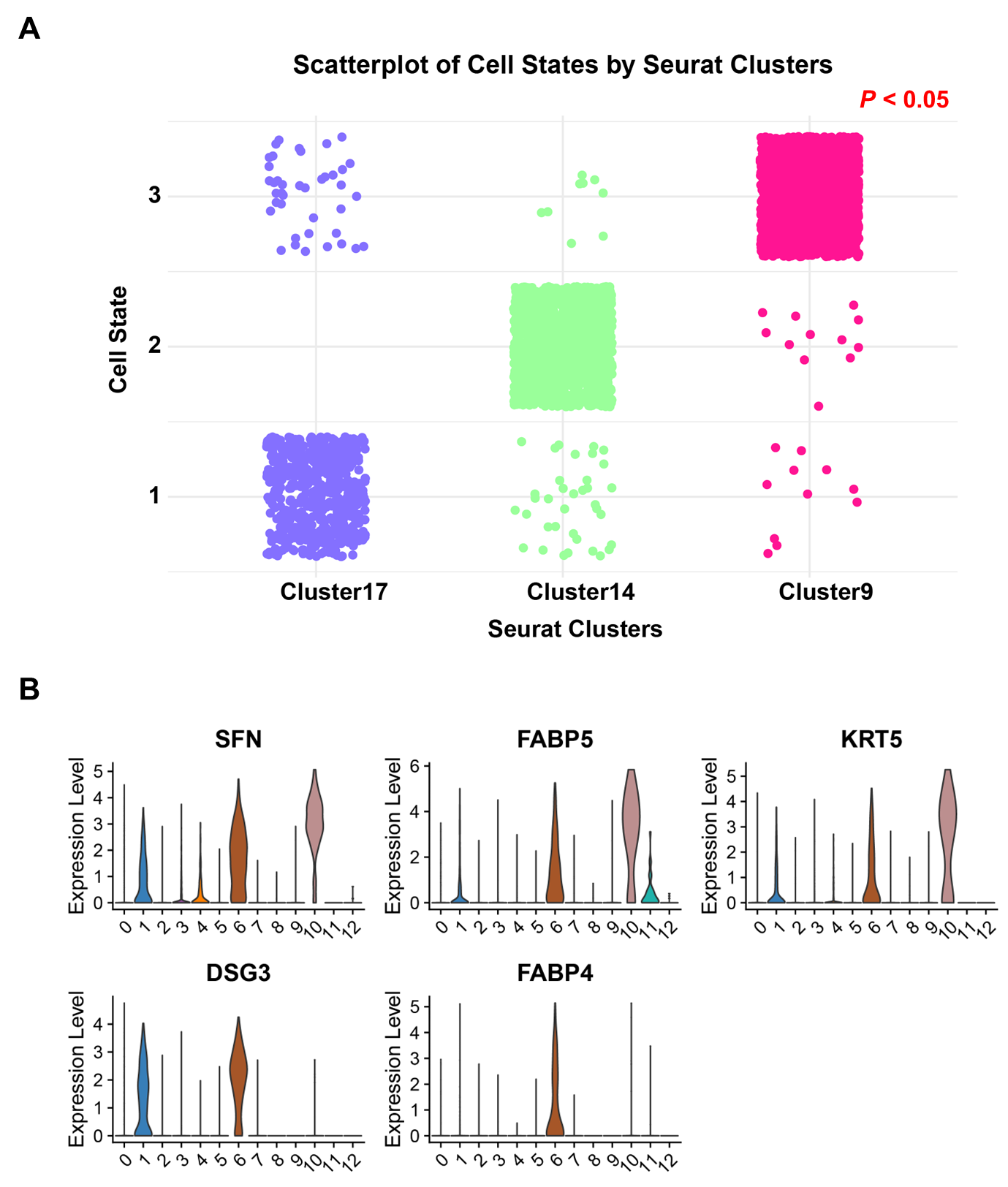
**

**Fig. S3. A** Scatterplot of cell states by seurat clusters. **B** Violin plots displayed the expression of malignant cell markers (*DSG3*, *FABP4*, *SFN*, *FABP5*, and *KRT5*) in epithelial cells.

**
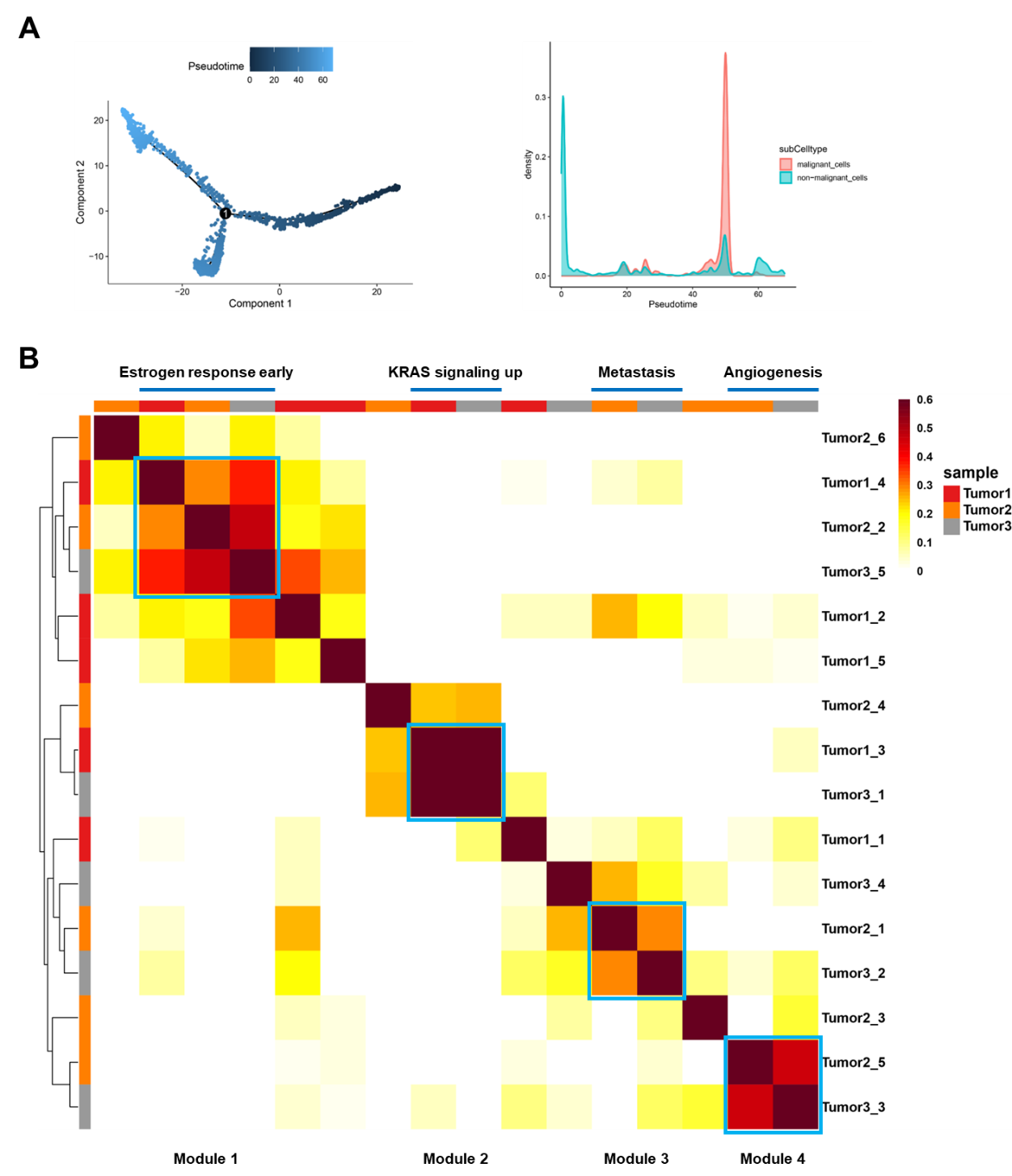
**

**Fig. S4. A** Trajectory analysis of the epithelial cells annotated by pseudotime (left) and density distribution of epithelial cells along the pseudotime trajectory (right). **B** Pairwise correlation clustering of intra-tumoral GEPs. 16 GEPs were derived by NMF from three tumors and formed four consensus modules (module 2 signature genes listed in Table S5), whose biological significance was predicted (top).

**
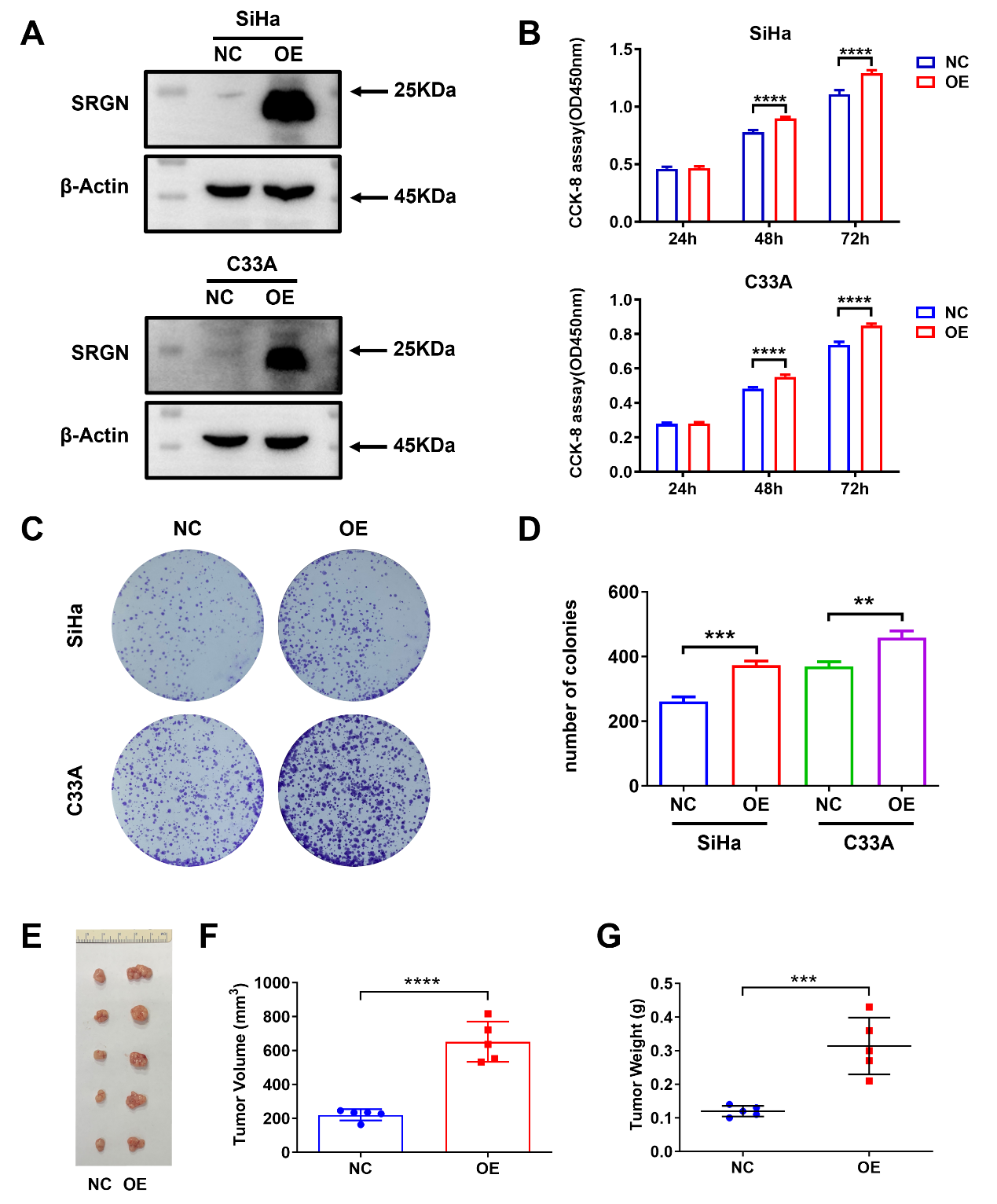
**

**Fig. S5.** **A** SRGN-overexpressing SiHa and C33A cells were constructed and confirmed via western blot. **B-D** The effect of SRGN overexpression on cell viability in SiHa and C33A cells was detected via CCK-8 assay (B) and colony formation (C-D). **E** Photograph of SRGN-overexpression Siha xenografts (right) relative to the control (left). **F**-**G** The final volume (F) and weight (G) of xenograft tumours in the SRGN-overexpression and control groups. OE: SRGN-overexpression, NC: normal control. ***P* < 0.01, ****P* < 0.001, *****P* < 0.0001.

**
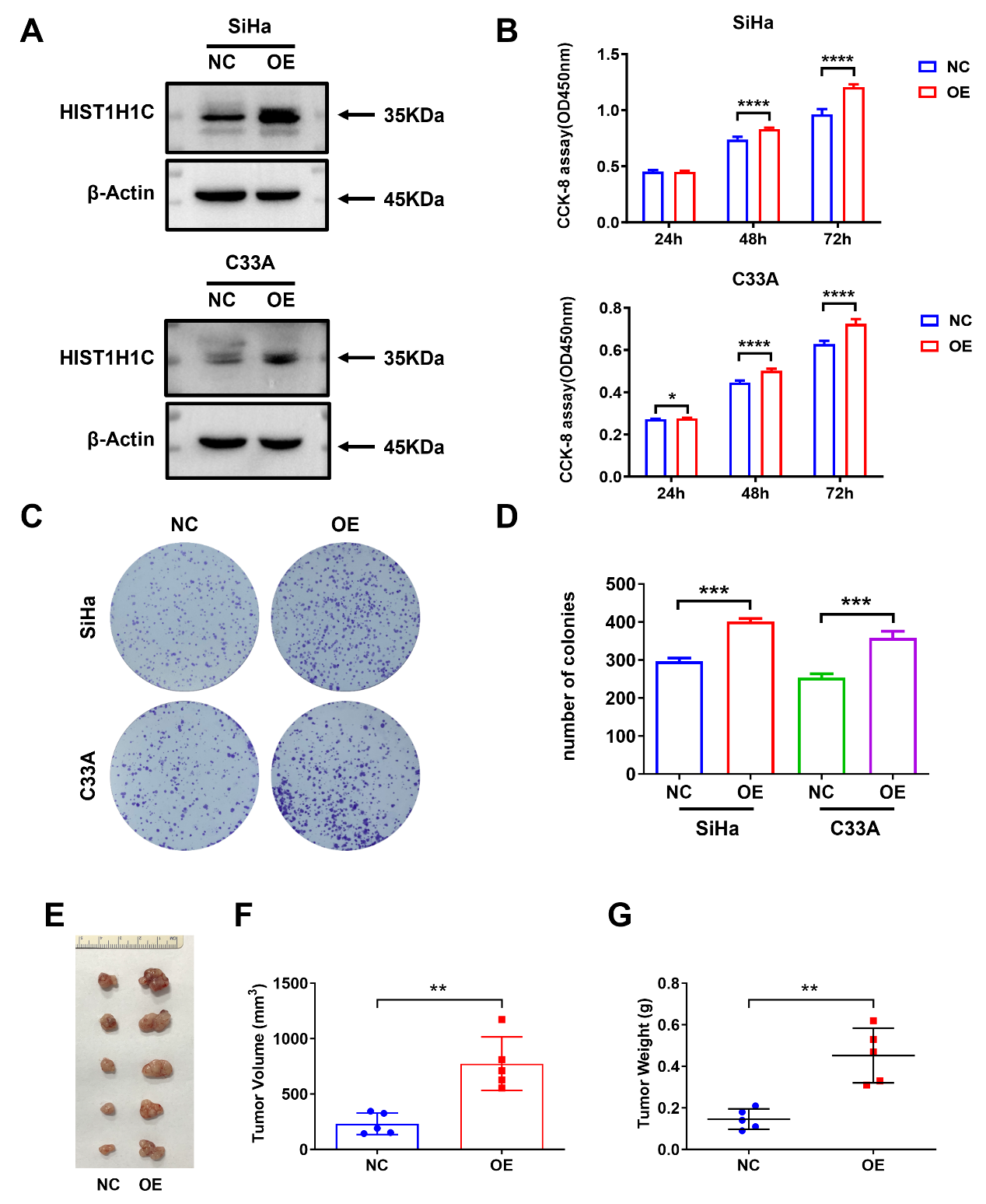
**

**Fig. S6.** **A** HIST1H1C-overexpressing SiHa and C33A cells were constructed and confirmed via western blot. **B-D** The effect of HIST1H1C overexpression on cell viability in SiHa and C33A cells was detected via CCK-8 assay (B) and colony formation (C-D). **E** Photograph of HIST1H1C-overexpression Siha xenografts (right) relative to the control (left). **F**-**G** The final volume (F) and weight (G) of xenograft tumours in the HIST1H1C-overexpression and control groups. OE: HIST1H1C-overexpression, NC: normal control. **P* < 0.05, ***P* < 0.01, ****P* < 0.001, *****P* < 0.0001.


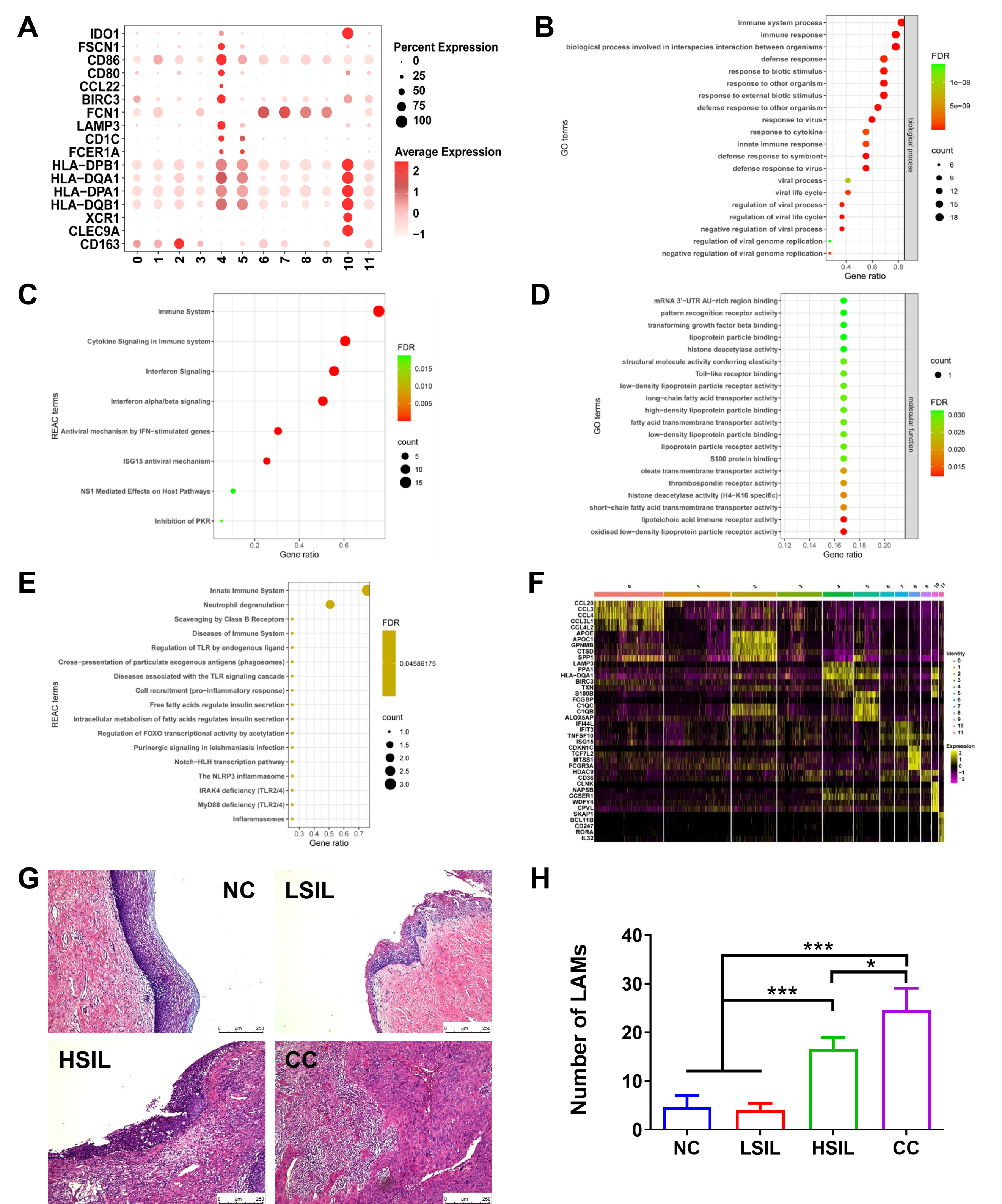


**Fig. S7. A** Expression of myeloid cell-specific markers across different clusters. The dot size is proportional to the fraction of marker-expressing cells in the group. **B**-**C** The GO Analysis and the REAC terms Analysis of subcluster 7 in myeloid cells. **D**-**E** The GO Analysis and the REAC terms Analysis of subcluster 9 in myeloid cells. **F** Heatmap showing the differentially expressed genes of myeloid cells across cluster, with top genes indicated. **G** The H&E staining of NC, LSIL, HSIL, and CC tissues. **H** The number of LAMs. **P* < 0.05, ****P* < 0.001.

**
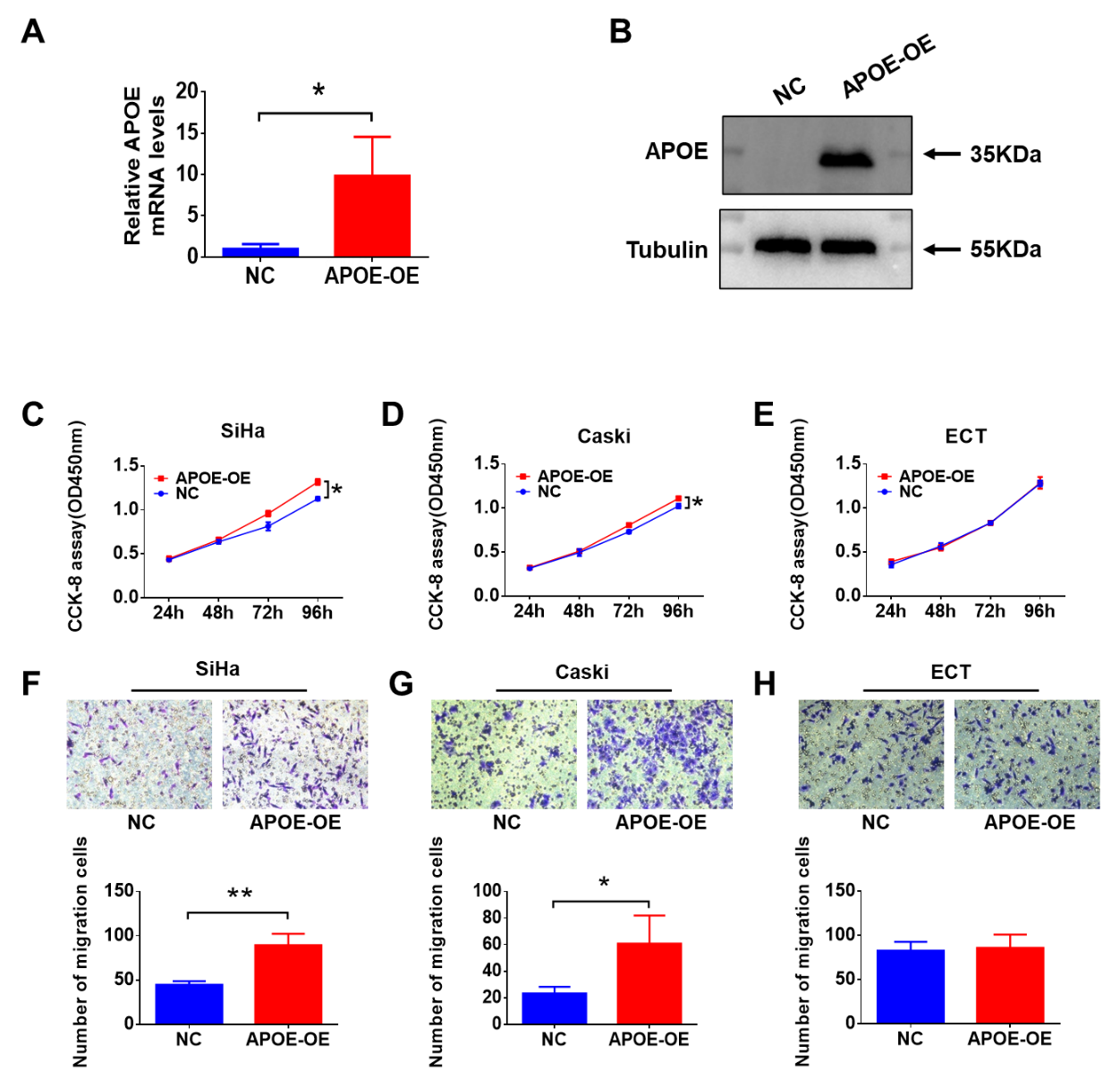
**

**Fig. S8. A** q-PCR analysis of APOE overexpression efficacy in THP-1 cells. **B** Western blot analysis of APOE overexpression efficacy in THP-1 cells. **C**-**E** CCK8 assay of SiHa, Caski, ECT cells with conditioned medium (CM) in different groups, respectively. **F**-**H** Transwell assay of SiHa, Caski, ECT cells cultured with the CM from APOE-OE macrophages and NC macrophages, respectively. **P* < 0.05, ***P* < 0.01.

**
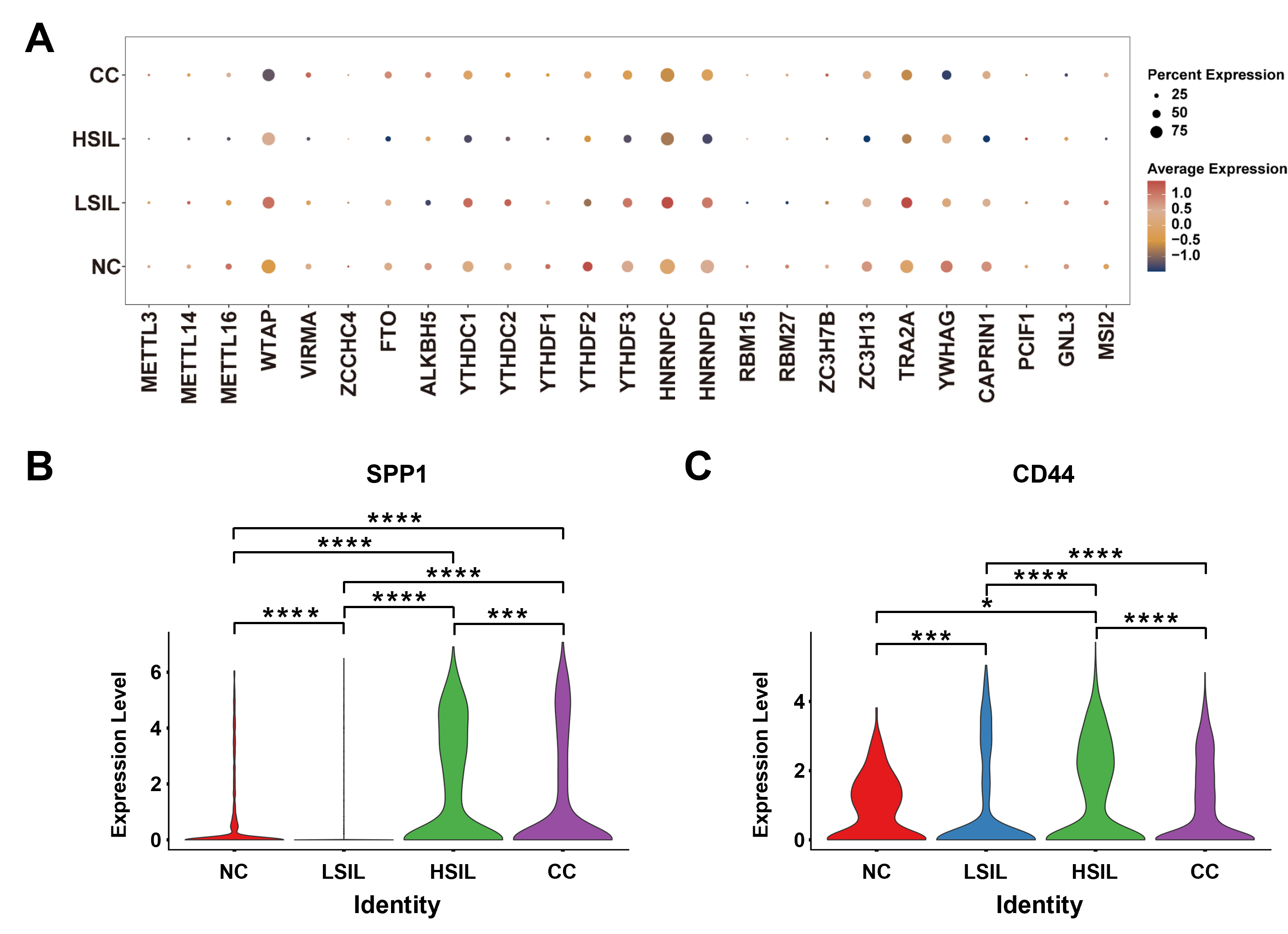
**

**Fig. S9. A** The expression of m6A methylation associated RNA transcripts of macrophage in NC, LSIL, HSIL, and CC group. **B-C** The level of *SPP1* in macrophages and *CD44* in epithelial cells in NC, LSIL, HSIL, and CC groups. **P* < 0.05, ****P* < 0.001, *****P* < 0.0001.
