# Supplementary figures and images for: Single-cell RNA sequencing of cervical exfoliated cells reveals potential biomarkers and cellular pathogenesis in cervical carcinogenesis
Source: Cell Death Dis. 2024 Feb 12;15(2):130. doi: 10.1038/s41419-024-06522-y (PMC10861450; doi:10.1038/s41419-024-06522-y)

**Fig. S5A**

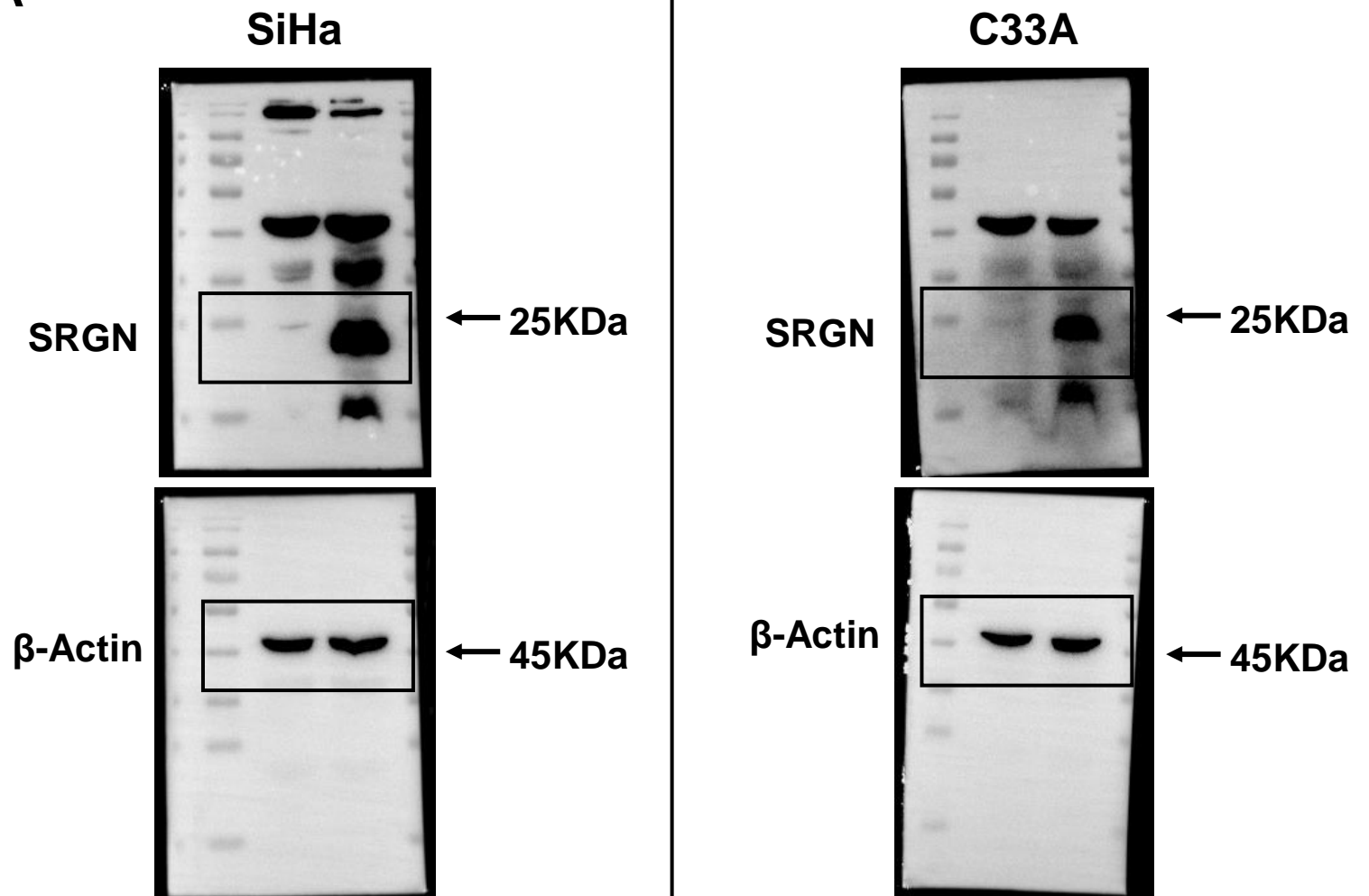

**Fig. S6A**

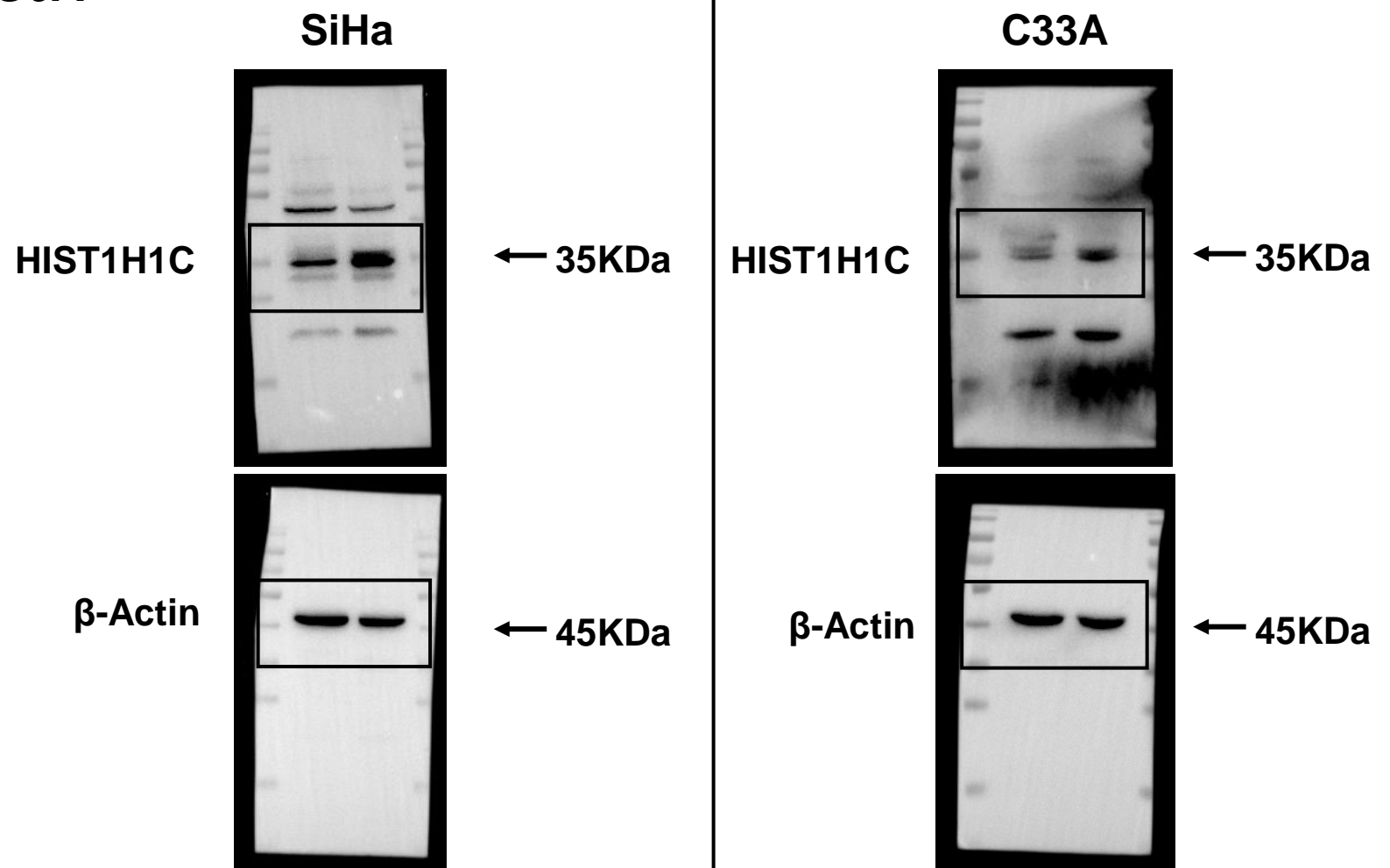

**Fig. S8B**

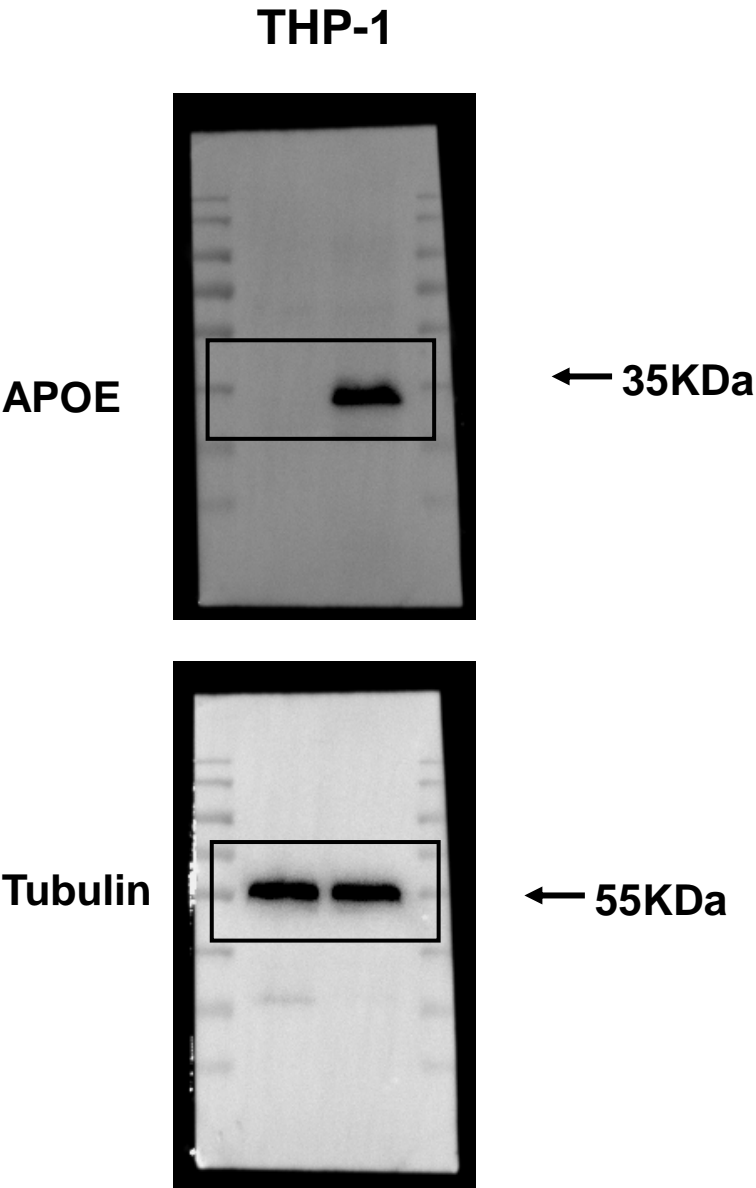

Supplement: Supplementary file 7 — Full and unccropped western blots [file 41419_2024_6522_MOESM7_ESM.pdf]
